# Pollen-expressed methionine synthases in *Nicotiana tabacum* regulate pollen development and germination

**DOI:** 10.1093/plphys/kiag572

**Published:** 2026-08-04

**Authors:** Jie Shu, Han Hu, Keliang Tao, Cai Feng, Zhuo Wei, Jiayuan Chang, Xiaojun Qin, Gonglin Chen, Han Wu, Gang Deng, Yongzhi Niu, Mingqiao Wang, Yunye Zheng, Suiyun Chen, Jugou Liao

**Affiliations:** School of Ecology and Environmental Sciences, Yunnan University, Biocontrol Engineering Research Center of Crop Diseases & Pests, State Key Laboratory of Vegetation Structure, Function and Construction (VegLab), Ministry of Education Key Laboratory for Transboundary Ecosecurity of Southwest China, Kunming, Yunnan 650500, China; School of Ecology and Environmental Sciences, Yunnan University, Biocontrol Engineering Research Center of Crop Diseases & Pests, State Key Laboratory of Vegetation Structure, Function and Construction (VegLab), Ministry of Education Key Laboratory for Transboundary Ecosecurity of Southwest China, Kunming, Yunnan 650500, China; School of Life Sciences, Yunnan University, Kunming, Yunnan 650500, China; School of Ecology and Environmental Sciences, Yunnan University, Biocontrol Engineering Research Center of Crop Diseases & Pests, State Key Laboratory of Vegetation Structure, Function and Construction (VegLab), Ministry of Education Key Laboratory for Transboundary Ecosecurity of Southwest China, Kunming, Yunnan 650500, China; School of Ecology and Environmental Sciences, Yunnan University, Biocontrol Engineering Research Center of Crop Diseases & Pests, State Key Laboratory of Vegetation Structure, Function and Construction (VegLab), Ministry of Education Key Laboratory for Transboundary Ecosecurity of Southwest China, Kunming, Yunnan 650500, China; School of Ecology and Environmental Sciences, Yunnan University, Biocontrol Engineering Research Center of Crop Diseases & Pests, State Key Laboratory of Vegetation Structure, Function and Construction (VegLab), Ministry of Education Key Laboratory for Transboundary Ecosecurity of Southwest China, Kunming, Yunnan 650500, China; School of Ecology and Environmental Sciences, Yunnan University, Biocontrol Engineering Research Center of Crop Diseases & Pests, State Key Laboratory of Vegetation Structure, Function and Construction (VegLab), Ministry of Education Key Laboratory for Transboundary Ecosecurity of Southwest China, Kunming, Yunnan 650500, China; School of Ecology and Environmental Sciences, Yunnan University, Biocontrol Engineering Research Center of Crop Diseases & Pests, State Key Laboratory of Vegetation Structure, Function and Construction (VegLab), Ministry of Education Key Laboratory for Transboundary Ecosecurity of Southwest China, Kunming, Yunnan 650500, China; School of Ecology and Environmental Sciences, Yunnan University, Biocontrol Engineering Research Center of Crop Diseases & Pests, State Key Laboratory of Vegetation Structure, Function and Construction (VegLab), Ministry of Education Key Laboratory for Transboundary Ecosecurity of Southwest China, Kunming, Yunnan 650500, China; School of Ecology and Environmental Sciences, Yunnan University, Biocontrol Engineering Research Center of Crop Diseases & Pests, State Key Laboratory of Vegetation Structure, Function and Construction (VegLab), Ministry of Education Key Laboratory for Transboundary Ecosecurity of Southwest China, Kunming, Yunnan 650500, China; Seed Engineering Technology Center, Yuxi Zhongyan Tobacco Seed CO., Ltd., Yuxi, Yunnan 653100, China; Abmart (Shanghai) Co., Ltd., Shanghai 200033, China; Yunnan Academy of Tobacco Agricultural Sciences, Kunming, Yunnan 650021, China; School of Ecology and Environmental Sciences, Yunnan University, Biocontrol Engineering Research Center of Crop Diseases & Pests, State Key Laboratory of Vegetation Structure, Function and Construction (VegLab), Ministry of Education Key Laboratory for Transboundary Ecosecurity of Southwest China, Kunming, Yunnan 650500, China; School of Ecology and Environmental Sciences, Yunnan University, Biocontrol Engineering Research Center of Crop Diseases & Pests, State Key Laboratory of Vegetation Structure, Function and Construction (VegLab), Ministry of Education Key Laboratory for Transboundary Ecosecurity of Southwest China, Kunming, Yunnan 650500, China

## Abstract

Methionine synthases (MetEs) are essential for plant metabolism and are highly expressed during reproductive processes. Despite their importance, the subcellular localization of MetEs and their specific roles and regulatory mechanisms in plant reproduction remain unclear. To address these knowledge gaps, we conducted subcellular localization and phylogenetic analyses of MetEs in the model species tobacco (*Nicotiana tabacum*). Additionally, we explored their functions through morphological, metabolomic, and transcriptomic analyses. The results showed that 6 NtMetEs belonging to dicot subclade 2 (DS2) localized in the endoplasmic reticulum, nucleus, and cytosol, while NsylMetE4 of DS1 with a transit peptide localized to the cytosol and nucleus. Knockout of 4 *NtMetE*s abundantly expressed in anthers reduced the pollen germination ratio by an average of 45.15% and methionine (Met) and S-adenosylmethionine (Sam) levels by 32.55% and 47.58%, respectively. Exogenous Met and Sam partially restored pollen germination in *mete1234*, and Met mainly rescued germination of pollen with normal size both in vivo and in vitro. In *mete1234* pollen, sucrose, starch, and energy metabolism were impaired, accompanied by reduced amyloplast accumulation and increased intine deposition. These results indicated that the localization of MetEs was diverse, and the transit peptide did not necessarily determine their plastid localization. *NtMetE1-4* synergistically regulated pollen development and germination through modulating carbohydrate metabolism and amyloplast deposition. These findings expand our understanding of MetEs localization, Met synthesis, and pollen fertility regulation, offering implications for plant reproduction.

## Teaser

Methionine-producing enzymes support pollen fertility by coordinating energy and carbohydrate metabolism during development, revealing how cellular location shapes reproductive success in plants.

## Introduction

Pollen development and germination enable double fertilization in flowering plants (Zhao et al. 2023). Several factors, including cell division, tapetum, pollen wall, and amyloplast development, significantly influence pollen activity (Ma et al. 2021). The tapetum provides nutrients and enzymes for microspore development and initiates programmed cell death, and materials for pollen wall formation (Wei and Ma, 2023). The pollen wall, comprising the exine and intine layers, is essential for maintaining pollen morphology and function (Ma et al. 2021). Starch bodies, or amyloplasts, are important for energy storage (Biswas and Chaudhuri 2024). Despite extensive insights into pollen development and fertility regulation, the roles of numerous pollen highly expressed genes, especially the methionine synthases (MetEs) family are still not well understood.

Methionine synthases, specifically cobalamin-independent 5-methyltetrahydropteroyltriglutamate-homocysteine methyltransferase (MetE) in plants, catalyze the methylation of homocysteine to methionine (Silva Rody and de Oliveira 2018). Then the cytosol-localized methionine adenosyltransferases (SAMS) convert Met into S-adenosylmethionine (Sam) (Meng et al. 2018; Huang et al. 2022). Sam further donates its methyl group during various methylation reactions, and subsequently forms S-adenosylhomocysteine (SAH) through the action of S-adenosylhomocysteine hydrolase (SAHH) (Yan et al. 2019). Research on the subcellular localization of these enzymes is vital for understanding where Met synthesis and metabolism occur. The MetEs of dicots include those from dicot subclade 1 (DS1) and dicot subclade 2 (DS2) (Silva Rody and de Oliveira 2018). Members of DS1 usually contain a transit peptide and are likely targeted to chloroplast, while DS2 members frequently lack transit peptides and are predicted to remain in the cytosol (Silva Rody and de Oliveira 2018). However, the role of the transit peptide in determining the subcellular localization of MetEs and the site where Met synthesis occurs requires further investigation.

Met synthesis and metabolism genes, especially *MetE*s, are widely expressed during plant reproduction (Ravanel et al. 1998). In *Chlamydomonas reinhardtii*, the transcript level of the *MetE* increases upon flagellar adhesion during gamete activation (Kurvari et al. 1995). In *Lotus japonicus*, *MetE* is also highly expressed in immature and mature anthers (Endo et al. 2002). In tobacco (*Nicotiana tabacum*), MetE localizes to Golgi-derived secretory vesicles concentrated in the apical region of pollen tubes, suggesting a role in pollen tube growth (Moscatelli et al. 2005). In *Arabidopsis*, Met accumulation inhibits pollen germination in the *sams3* mutant (Chen et al. 2016). Additionally, *AtMetE1* is highly expressed in flowers, and plays a key role in DNA and histone methylation (Yan et al. 2019). The inability to obtain homozygous *AtMetE1* knockout lines suggests that *AtMetE1* is essential for reproduction and development in *Arabidopsis* (Yan et al. 2019). Collectively, these findings indicate that MetEs might play an important role in plant reproduction. However, their specific functions and regulatory mechanism in pollen development and germination remain to be elucidated.

In this study, tobacco (*N. tabacum*) was used as a model system to investigate the role and regulatory mechanism of *NtMetE*s in pollen development and germination, as well as the subcellular localization of MetEs and the sites of Met synthesis. By screening the monoclonal antibodies (NtmAbs) against *Nicotiana* reproductive tissues (Chen et al. 2020), we identified the anther abundantly expressed NtMetEs at the protein level, and determined their distribution and expression during pollen/anther development by in situ immunofluorescence (IF) and transcriptional analysis. We genetically verified the function of *NtMetE1-4* in pollen development and germination, and explored how they function through morphological, metabolomic, and transcriptomic analyses. We found that *NtMetE1*-*4* synergistically regulated anther/pollen development, and pollen germination by regulating carbohydrate metabolism and amyloplast deposition. Furthermore, phylogenetic and subcellular localization analyses revealed that the N-terminal transit peptide of MetEs did not necessarily determine their chloroplast localization, and we found that NtMetEs were localized in the nucleus, endoplasmic reticulum (ER), and cytosol. These findings expand our understanding of MetEs localization and Met synthesis, and the mechanisms regulating pollen development, germination, and plant reproduction.

## Results

### NtMetEs are abundantly expressed in the anther wall and maturing pollen

To identify proteins (especially NtMetEs) that are highly expressed in the anther, we screened DEPs in the anthers and stigmas of *Nicotiana* using monoclonal antibody slides against reproductive tissues (NtmAbs) (Chen et al. 2020). Among these DEPs, 16 proteins abundantly expressed in anthers were identified via LC‒MS/MS (Table S1), and their expression profiles were further validated by Western blot (WB) analysis (Figure S1a and b). These DEPs included SAHH, MetE, and SAMS, which regulate Met and Sam metabolism (Figure S1b), indicating that this metabolic pathway may play an important role in the anther/pollen development.

To explore the role of *NtMetE*s in anther/pollen development, we identified *NtMetE* family members in the allotetraploid genome of tobacco (*N. tabacum*) and its ancestor. Genome searches and sequence analyses revealed 6 *NtMetE*s in the allotetraploid genome (*NtMetE1*, *2*, *3*, *4*, *5*, and *6*) (Table S2), whereas 4 *MetE*s were identified in each of its diploid ancestors, *Nicotiana sylvestris* (*NsylMetE1*, *2*, *3*, and *4*) and *Nicotiana tomentosiformis* (*NtomMetE1*, *2*, *3*, and *4*) (Table S2).

To further identify the *NtMetE*s members abundantly expressed in anthers, we examined the transcript levels of *NtMetE1-6* in the roots, stems, leaves, anthers, and pistils. The results revealed that the expression levels of 6 *NtMetE*s were highest in anthers (Figure S1c), with *NtMetE2* showing the highest transcript levels, followed by *NtMetE1* and *NtMetE3*. Furthermore, the expression levels of *NtMetE1*, *NtMetE2*, and *NtMetE3* increased after pollination, possibly in the pollen or the pistil itself. In contrast, *NtMetE4*, *5*, *6* exhibited relatively low expression levels (Figure S1c). These data reveal that *NtMetE1*, *2*, *3* may be more important in anthers and pollen than *NtMetE4*, *5*, *6*.

**Figure 1:**
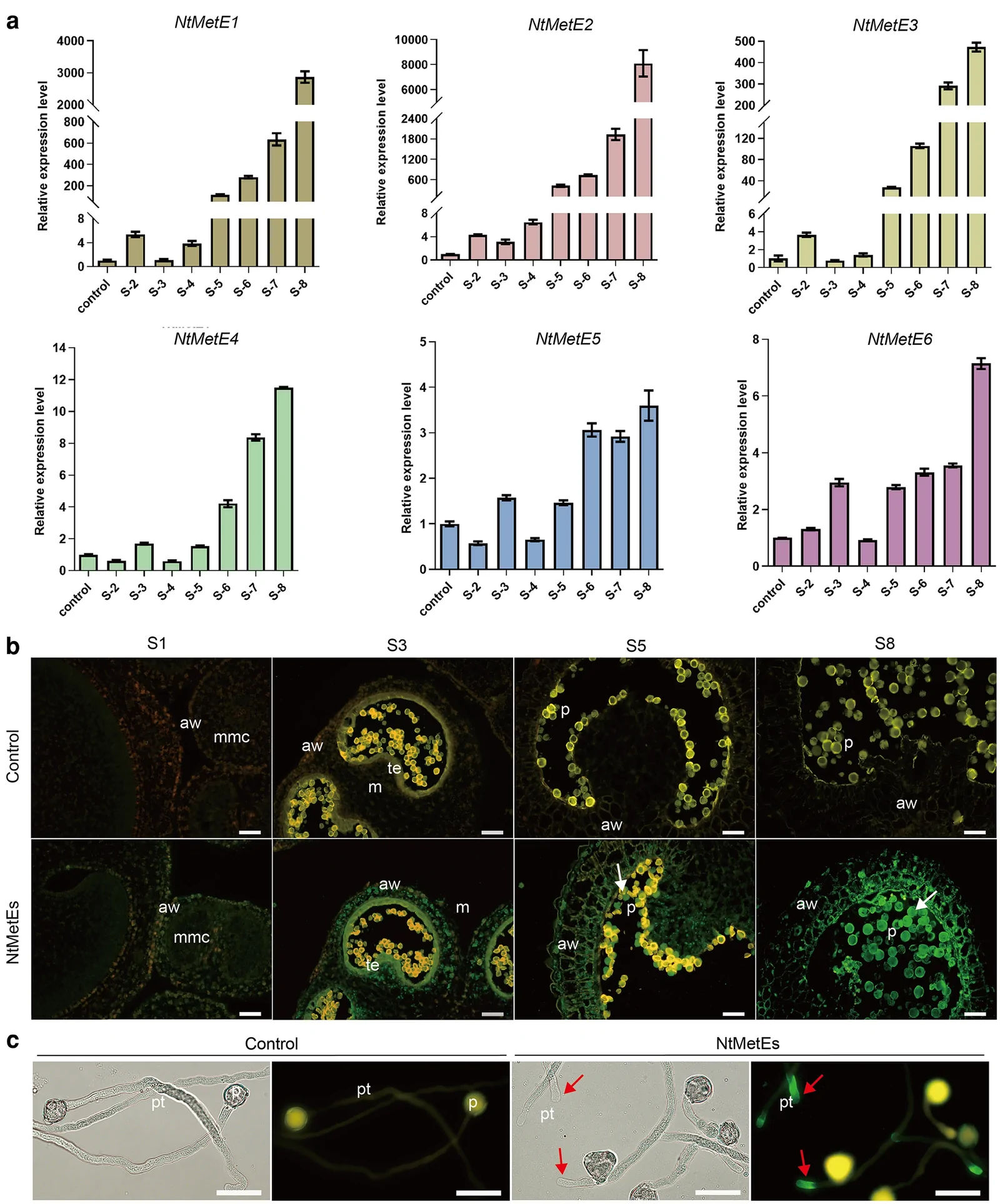
NtMetEs are abundantly expressed in anther wall, maturing pollen, and pollen tube tips. a) RT-qPCR analysis of NtMetEs expression during anther development. Anther at stage 1 was used as the control. b, c) Immunofluorescence (IF) analysis on the expression and distribution of NtMetEs in developing anther and in vitro growing pollen tubes using mouse monoclonal anti-NtMetEs antibody (N49). Primary antibody is replaced with 1× PBS buffer in control. Green fluorescence represents NtMetEs; yellow fluorescence may result from excess secondary antibody incubation. aw, anther wall; te, tapetum; p, pollen; m, microspore; mmc, microspore mother cell; pt, pollen tube. Red arrows indicate pollen tube tips. Error bars represent SD from 3 technical replicates. Scale bars = 50 μm.

To determine the role of NtMetEs in the sporophyte or gametophyte, and the developmental stages they function, we analyzed their expression and distribution during anther development via reverse transcription quantitative PCR (RT-qPCR) and IF assays. The expression levels of *NtMetE*s increased from stages 5 or 6 (Fig. 1a), when binucleate pollen began to mature (Liao et al. 2020a, 2020b), and peaked in the mature anthers (Fig. 1a). *NtMetE1*, *2*, *3* exhibited higher expression levels than *NtMetE4*, *5*, *6* (Fig. 1a).

IF analysis revealed that the green fluorescence of NtMetEs was detected in the anther wall at all developmental stages and at the tapetum of stage 3, while it occurred in pollen from stage 5 (Fig. 1b), with the strongest intensity observed at stage 8 (Fig. 1b). Furthermore, the green fluorescence was also detected in pollen tube tips (Fig. 1c). These IF results show that *NtMetE1*, *2*, *3* are expressed in maturing pollen and pollen tube tips, suggesting their potential role in pollen development and pollen tube growth.

### The N-terminal transit peptide of NsylMetE4 affects its subcellular localization

**Figure 2:**
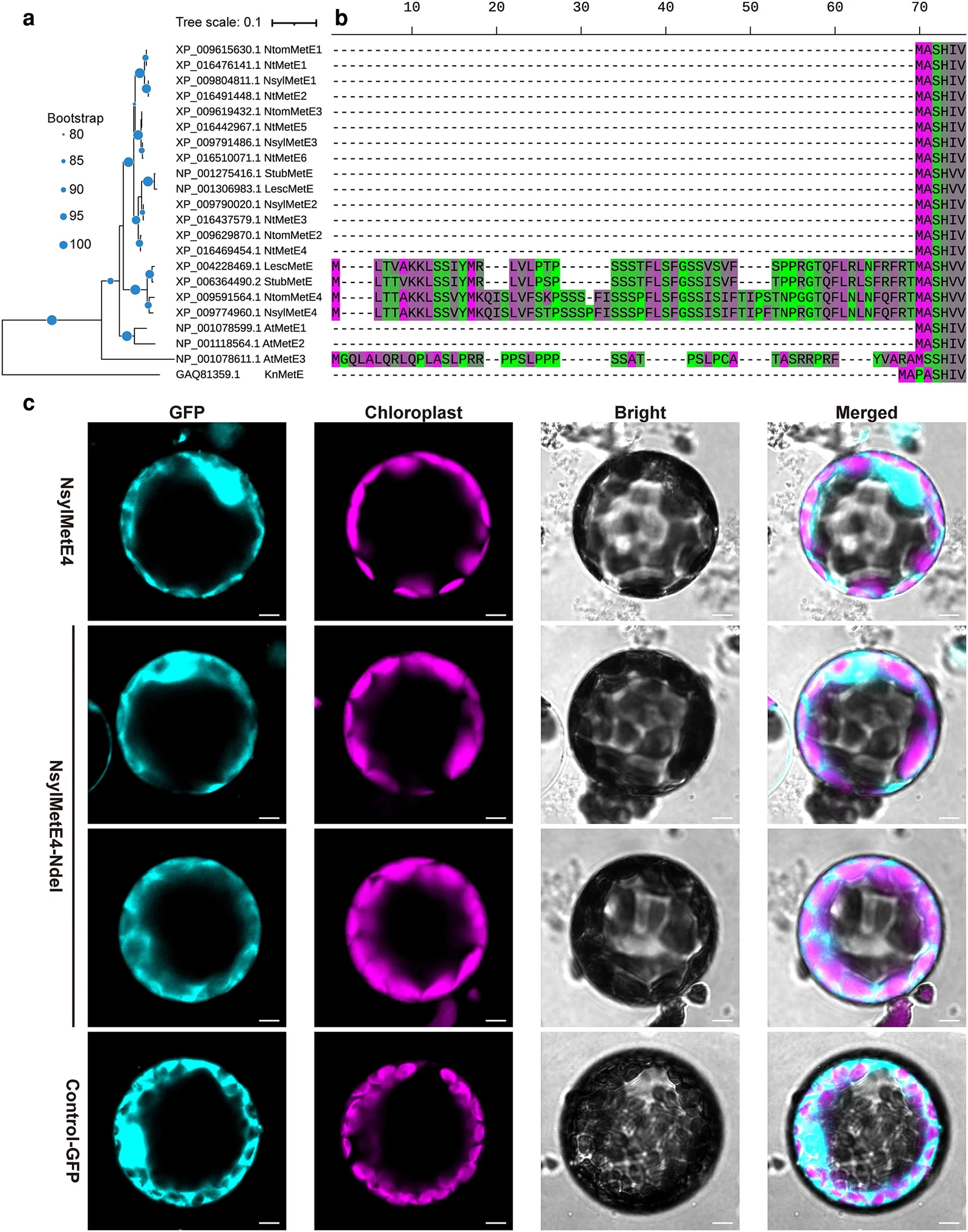
Sequence and phylogenetic analysis of MetEs, and subcellular localization of NsylMetE4. aA and b) Phylogenetic tree of MetEs from *N. tabacum* (NtMetEs), *N. tomentosiformis* (NtomMetEs), *N. sylvestris* (NsylMetEs), *A. thaliana*, *S. tuberosum*, and *S. lycopersicum*, with one MetE of *K. nitens* (GAQ81359.1) as the outgroup. Tree scale bar indicates 0.1 substitutions per site. c) Subcellular localization of NsylMetE4 and NsylMetE4-Ndel (lacking N-terminal transit peptide). Scale bars = 10 μm. Panel b shows the MAFFT multiple sequence alignment, with signal peptide regions extracted and visualized.

To determine the phylogenetic relationship of NtMetE1-6 with their ancestral homologs in *N. sylvestris* and *N. tomentosiformis*, we further performed phylogenetic analyses among NtMetEs, NtomMetEs, and NsylMetEs. The results revealed that NtMetE1, 4 and 5 were homologous to NtomMetE1, 2 and 3, respectively, whereas NtMetE 2, 3 and 6 clustered with NsylMetE1, 2 and 3 (Fig. 2a). The protein sequences similarity between NtMetE1 and 2, NtMetE3 and 4, or NtMetE5 and 6 exceeded 98% (Table S3), suggesting that *NtMetE1*, *2*, *NtMetE3*, *4*, and *NtMetE5*, *6* represent homologous gene pairs in allotetraploid *N. tabacum.* Additionally, corresponding orthologs of the *NtomMetE4* and *NsylMetE4* genes were absent from the genome of *N. tabacum* (Fig. 2a).

The N-terminal transit peptide determines the subcellular localization of MetEs, and we further performed sequence analysis to explore whether such an N-terminal transit peptide is present in these *Nicotiana* MetEs. The results revealed that the N-terminal peptide was absent in NtMetE1-6, NsylMetE1-3, and NtomMetE1-3, all of which clustered into clade 2 (Fig. 2a). In contrast, NtomMetE4 and NsylMetE4 possess an N-terminal transit peptide, similar to MetEs from *Solanum lycopersicum* (LescMetE), *Solanum tuberosum* (StubMetE), and AtMetE3 (Fig. 2b). The transit peptide sequences between NtomMetE4 and NsylMetE4, as well as LescMetE and StubMetE, show high similarity. However, the transit peptide sequence similarity between these MetEs and AtMetE3 is low (Fig. 2b), indicating they may contribute to differential subcellular localizations of MetEs. NsylMetE4, NtomMetE4, StubMetE, and LescMetE clustered into clade 1 (Fig. 2a). These data collectively indicate that all NtMetEs lacking an N-terminal transit peptide belong to clade 2, and that the orthologous genes of NsylMetE4 and NtomMetE4 are absent from the genome of *N. tabacum* L. “K326.”

Since AtMetE3, which contains an N-terminal transit peptide, has been reported to localize to the chloroplast (Ravanel et al. 2004), we analyzed whether NsylMetE4 containing transit peptide exhibited similar subcellular localization. The results showed that NsylMetE4 was localized to the nucleus and cytosol (Fig. 2c). Moreover, deletion of the N-terminal transit peptide of NsylMetE4 weakened its nuclear localization (Fig. 2c), suggesting that the transit peptide contributes to the nuclear localization of NsylMetE4.

### NtMetEs are localized in ER, cytosol, and nucleus

**Figure 3:**
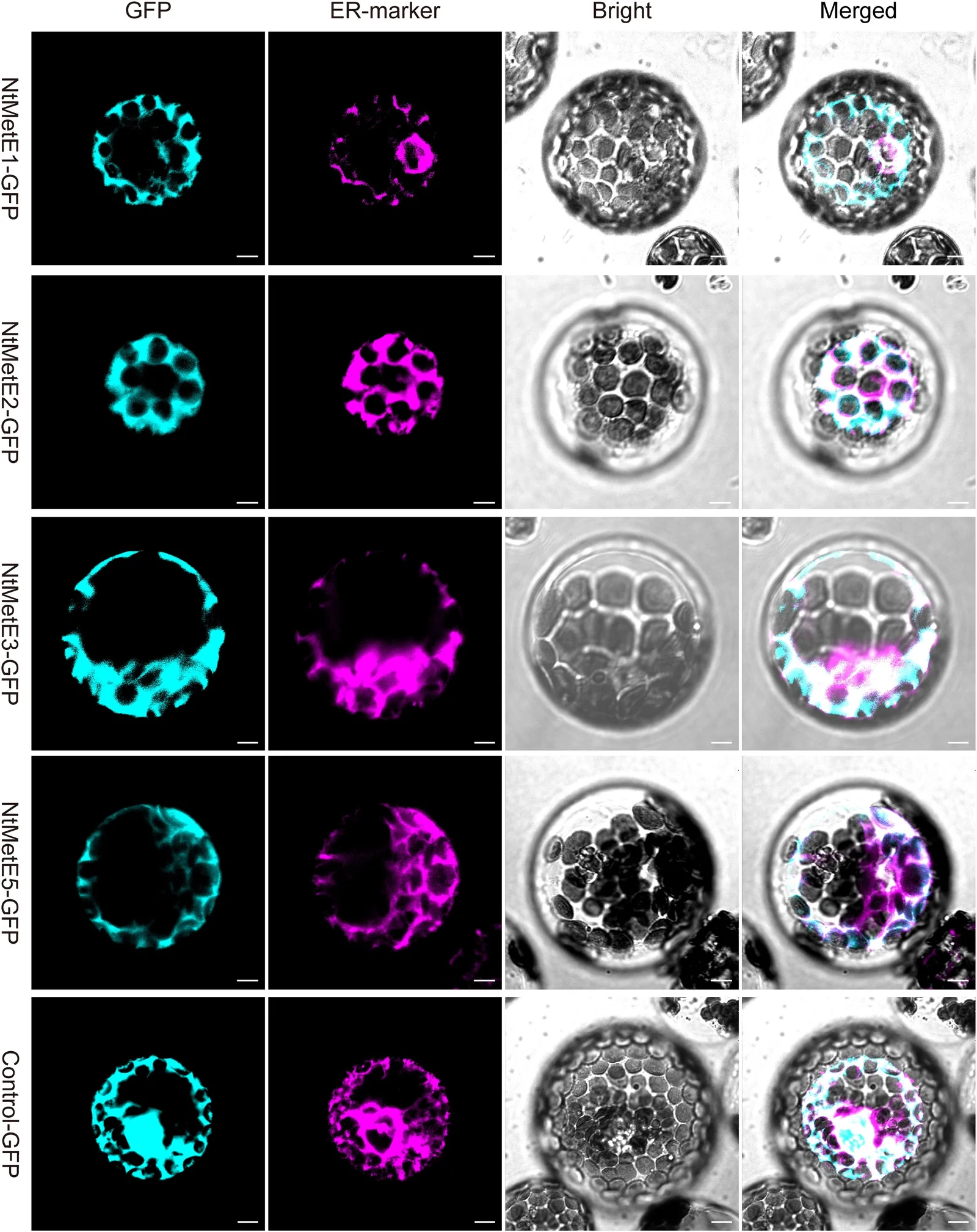
Subcellular localization of NtMetE1, NtMetE2, NtMetE3, and NtMetE5. Subcellular localization of NtMetE1, NtMetE2, NtMetE3, NtMetE5, and GFP in the protoplasts. The ER marker (SPER-mKate) used in this study was adopted on the basis of the reference (Zhao et al. 2024).

The subcellular localization of MetEs determines where Met synthesis occurs, and previous studies have reported that MetEs localize to the cytosol, chloroplasts, and Golgi-derived vesicles (Ravanel et al. 2004; Moscatelli et al. 2005), but the subcellular localization of NtMetEs remains unclear. Our data revealed that NtMetE1 was potentially localized in the ER (Figure S2a). Further co-localization analysis using an ER marker (SPER-mKate) showed that the green fluorescence of NtMetE1, NtMetE2, NtMetE3, and NtMetE5 extensively overlapped with the red fluorescence of SPER-mKate, confirming their ER localization (Fig. 3). Additionally, the green fluorescence of NtMetE1, NtMetE3 and NtMetE5 was also observed in the cytosol (Fig. 3), indicating their dual localization in both the ER and cytosol.

As partial SPER-mKate fluorescence in the protoplast did not display a typical ER pattern (Fig. 3), we further verified the localization of NtMetEs by transient expression assays in *Nicotiana* leaves (Fig. 3 and Figure S2). The results showed that the SPER-mKate fluorescence exhibited a typical ER pattern, and the green fluorescence of NtMetE1, NtMetE3, and NtMetE5 overlapped with the red fluorescence of SPER-mKate, while that of NtMetE2 also was observed in the nucleus (Fig. 3). These data collectively indicated that NtMetE1, NtMetE3 and NtMetE5 localized in the cytosol and ER, whereas NtMetE2 was targeted to the nucleus, cytosol, and ER.

Given that the localization of NtMetEs differs from that in *Arabidopsis*, we further examined the localization of cystathionine β-lyase (CBL) catalyzing the cystathionine to homocysteine (upstream step of Met synthesis), and the localization of SAMS and SAHH responsible for Met metabolism and recycling (Hesse et al. 2004). The results revealed that NtCBL2 was localized in chloroplasts (Figure S3), consistent with previous findings (Liu et al. 2019). NtSAHH4 was localized to the cell membrane, and NtSAHH7 was targeted to the nucleus and cytosol (Figure S3), similar to the localization pattern reported in *Arabidopsis* (Lee et al. 2012). Likewise, NtSAMS5 was detected in both the nucleus and cytosol, while NtSAMS4 was localized to the cell membrane (Figure S3).

### Knockout of *NtMetEs* causes more severe functional impairments in the male parts

According to the phylogenetic relationship and the high expression levels of *NtMetE1*, *2*, *3*, *4* in anther and pollen, we generated knockout mutants against them via CRISPR/Cas9 to investigate their function in anther and pollen. Double-knockout lines (*mete23*, *mete14*), triple-knockout lines (*mete124*, *mete123*, and *mete 12^+/−^34*), and a quadruple-knockout line (*mete1234*) were obtained. Detailed information on the mutated sequences was provided in Figure S4a and Table S4. Insertion or deletion of the target sites resulted in protein translation pre-termination, causing functional loss (Figure S4a). Sequence analysis confirmed that *NtMetE5* and *NtMetE6* were not edited in any of the mutants (Figure S4a).

To confirm the reduction in NtMetEs protein levels resulting from protein translation pre-termination, we conducted IF analysis on mature pollen and pollen tubes of *mete1234* using 2 monoclonal antibodies against NtMetEs (N21 and N49) (Chen et al. 2020). The results showed reduced NtMetEs green fluorescence levels in pollen grains and tube tips (Figure S4g and h), confirming that editing of *NtMetE*s decreased protein levels. This reduction may influence pollen development and function.

Knockout of *NtMetEs* resulted in smaller leaves (Figure S4b). In addition, the flowering of *mete124*, *mete134*, *mete12^+/−^34*, and *mete1234* was delayed (Figure S4b). The fruit weights of the double-knockout lines *mete23* and *mete14* changed insignificantly, whereas that of *mete124* decreased by 41.76% (Figure S4c to f). The fruit weights of *mete134*, *mete12^+/−^34*, and *mete1234* were the lowest, reducing by 79.39%, 78.38%, and 83.21%, respectively (Figure S4c and f). The weight and number of seeds per fruit reduced by 84.29% and 84.04% in *mete1234* (Figure S4d and e). These results reveal that *NtMetE1*, *2*, *3*, *4* redundantly regulated fruit weight, with *NtMetE3* playing a more predominant role than *NtMetE2*.

**Figure 4:**
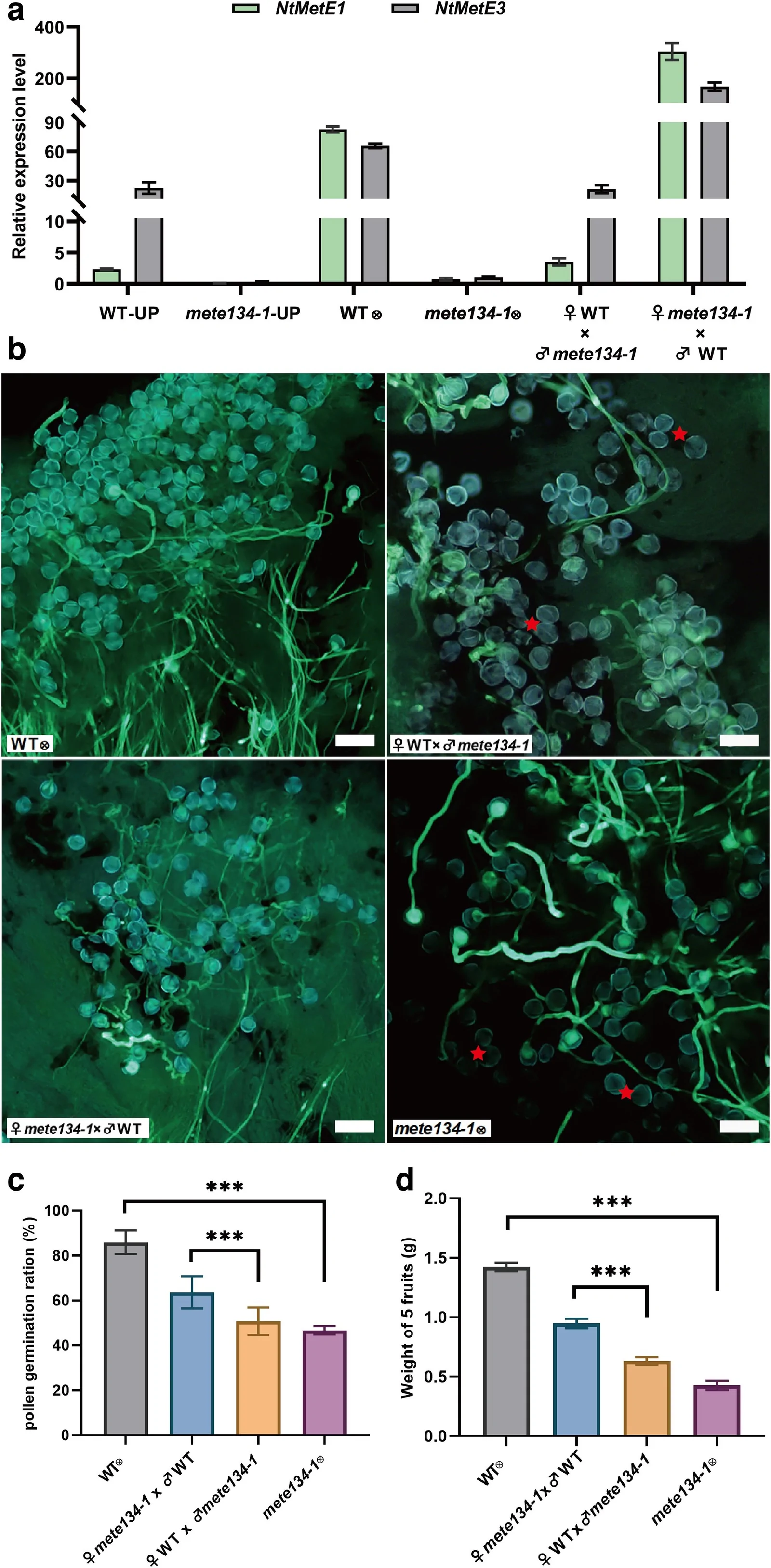
Reciprocal crossing between *mete134* and WT showed that *NtMetE*s functioned mainly in the paternal part. a) RT-qPCR analysis of NtMetE1 and NtMetE3 expression in pistils from reciprocal crosses between WT and mete134. UP, unpollinated pistil; ⊗, self-pollinated pistil. Error bars represent mean + SD from 3 technical replicates. b) Pistils from reciprocal crosses stained with aniline blue. Red stars mark ungerminated pollen grains. Scale bars = 50 μm (top), 200 μm (bottom). c) Pollen germination rates on reciprocally pollinated stigmas. Data are means ± SD from 3 biological replicates (Student's *t*-test, ***P < 0.001). d) Fruit weight after reciprocal crosses between *mete134* and WT (*n* = 5 fruits, Student's *t*-test, ***P < 0.001). Error bars represent SD from 3 technical replicates.

To investigate whether *NtMetE*s regulate fruit weight primarily through the paternal or maternal parts, we conducted reciprocal pollination (between WT and *mete134*), and analyzed the expression of *NtMetE*s. The data revealed that expression levels of *NtMetE1*, *3* were low in unpollinated pistils of WT and *mete134*, in *mete134* self-pollinated pistils, and in WT pistils pollinated by *mete134* (Fig. 4a). In contrast, their expression levels increased more than 43-fold in WT self-pollinated pistils and in *mete13* pistils pollinated by the WT pollen (Fig. 4a). These results indicate that the transcripts of *NtMetE1*,*3* detected in pollinated pistils were mainly from pollen and pollen tubes. Therefore, the reduction in fruit weight is mainly influenced by the *NtMetE*s expression in pollen.

Furthermore, the pollen germination ratio and fruit weight in ♀ WT × ♂ *mete134* were 12.93% and 31.68% lower than that of ♀ *mete134* × ♂ WT (Fig. 4b to d). Although the fruit weight and pollen germination ratio also decreased by 33.29% and 22.33% in the ♀*mete134* × ♂ WT group compared with the ♀ WT×♂ WT group (Fig. 4b to d). These results indicate that *NtMetE*s play a greater role in pollen than in pistils. Our subsequent research focused on how NtMetEs regulate pollen development and germination.

### *NtMetE1-4* redundantly regulate anther development and pollen germination

**Figure 5:**
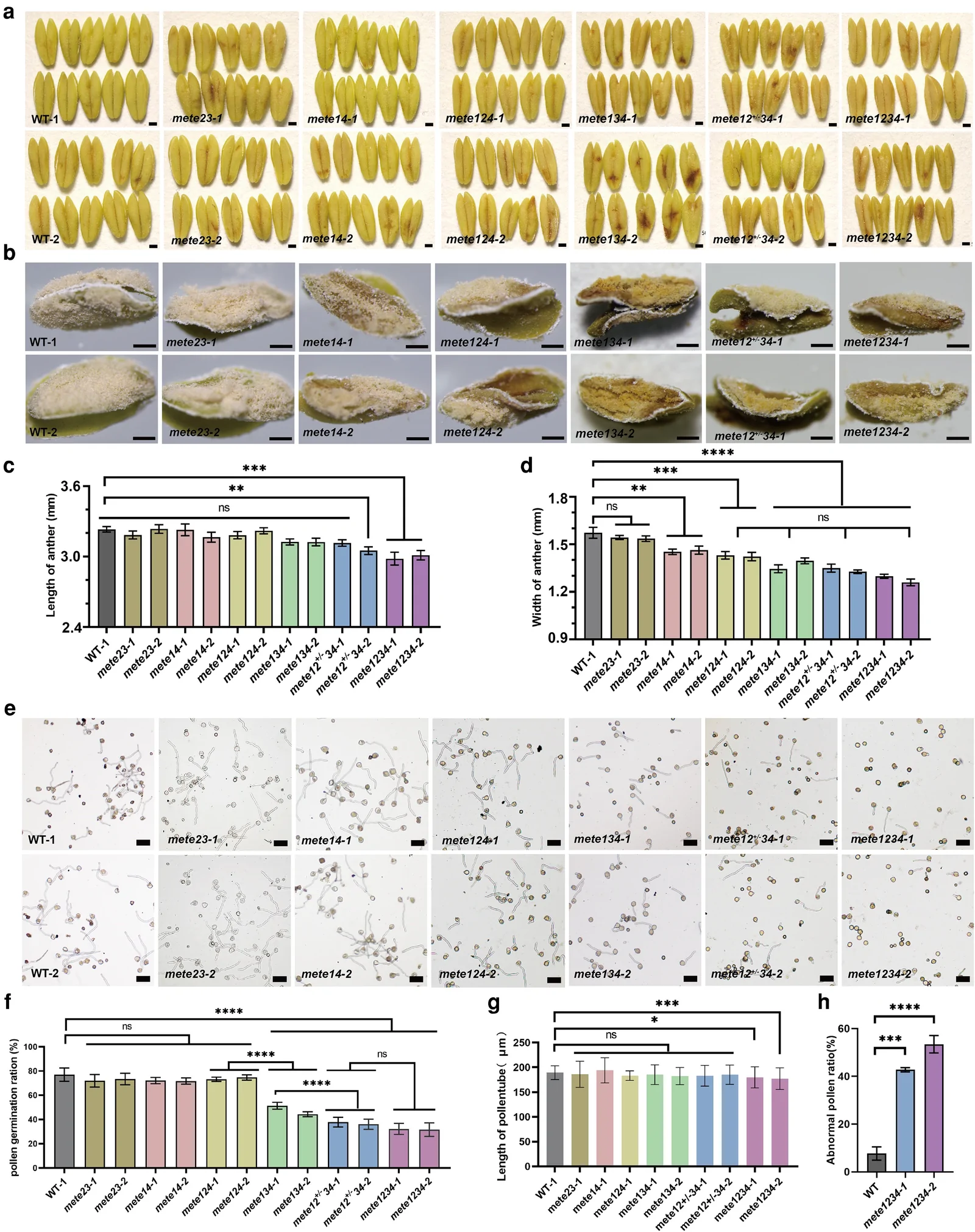
*NtMetE1, 2, 3,* and *4* redundantly regulate anther/pollen development and fruit weight. a) Morphology and size of anthers in WT and mutants. Scale bar = 0.5 mm. b) Morphology of dehiscent anthers. Scale bar = 2 mm. c, d) Quantification of anther width and length. Ten anthers were measured per mutant plant. e) In vitro pollen germination in WT and mutants. Pollen germination assays were repeated 3 times. Scale bar = 100 μm. f) Quantification of pollen germination rates shown in panel e (*n* = 500 grains). g) Quantification of pollen tube length of WT and mutants (*n* = 200 pollen tubes). h) Proportion of shriveled pollen in WT (*n* = 279) and *mete1234* (*n* = 413). Statistical analysis was performed using one-way ANOVA (ns, nonsignificant; *P < 0.05; **P < 0.01; ***P < 0.001; ****P < 0.0001. Error bars represent SD from 3 technical replicates).

To further explore the functional relationships among *NtMetE1-4* during anther and pollen development, we compared the anther size, pollen number, and pollen germination ratio among the double-, triple-, and quadruple-knockout mutants. The results revealed that the anther lengths of *mete12^+/−^34* and *mete1234* were 4.6% and 7.26% shorter than those of the WT (Fig. 5a and c). The anther width decreased the most in *mete134*, *mete12^+/−^34*, and *mete1234* (Fig. 5a and d), reduced by 12.87%, 14.94%, and 8.72%, respectively. Furthermore, anatomical observations revealed that the anthers of the triple- and quadruple-knockout lines were less plump (Fig. 5b). These results suggest that *NtMetE1-4* redundantly regulate anther development.

In vitro germination analysis revealed that the pollen germination ratio did not change significantly in *mete23*, *mete14*, or *mete124*, but was significantly reduced in *mete134*, *mete12^+/−^34*, and *mete1234* (Fig. 5e and f). The pollen germination ratio of *mete134* was 26.1% lower than that of *mete124*. The lowest germination ratios were detected in *mete12^+/−^34* and *mete1234*, reduced by 39.19% to 45.46% compared to WT (Fig. 5e and f). These results indicate that *NtMetE1-4* redundantly regulate pollen germination, with *NtMetE3* having a more pronounced impact than *NtMetE2*. The pollen tube length of *mete1234* also decreased by 10.78% (Fig. 5g), consistent with the expression of NtMetEs at the tips of growing pollen tubes (Fig. 1c). Collectively, *NtMetE*s regulate both pollen germination and tube growth, with their roles in pollen germination being more prominent than those in pollen tube elongation.

### *NtMetE*s modulate pollen starch deposition and wall formation

As anther size decreased in mutants, we further examined the anther structure of the mutants. Morphological observations revealed that the structure of anther wall and tapetum of *mete1234* were normal (Figure S5c), indicating that the reduced pollen germination ratio was not caused by structural abnormalities of the sporophyte of anther (Fig. 5a, c, and d).

The mature pollen germination ratio decreased significantly in mutants; we then identified the stages at which pollen viability decreased by fluorescein diacetate (FDA)/propidium iodide (PI) staining. The results revealed that the pollen viability of *mete1234* decreased by 10.9% at stage 6, by 59.81% at stage 8 (Figure S5a and b). These findings suggested that *NtMetE*s affect pollen development after the binucleate stage. The decline in pollen viability at stage 6 coincided with the accumulation of NtMetEs in the pollen (Figure S1b).

**Figure 6:**
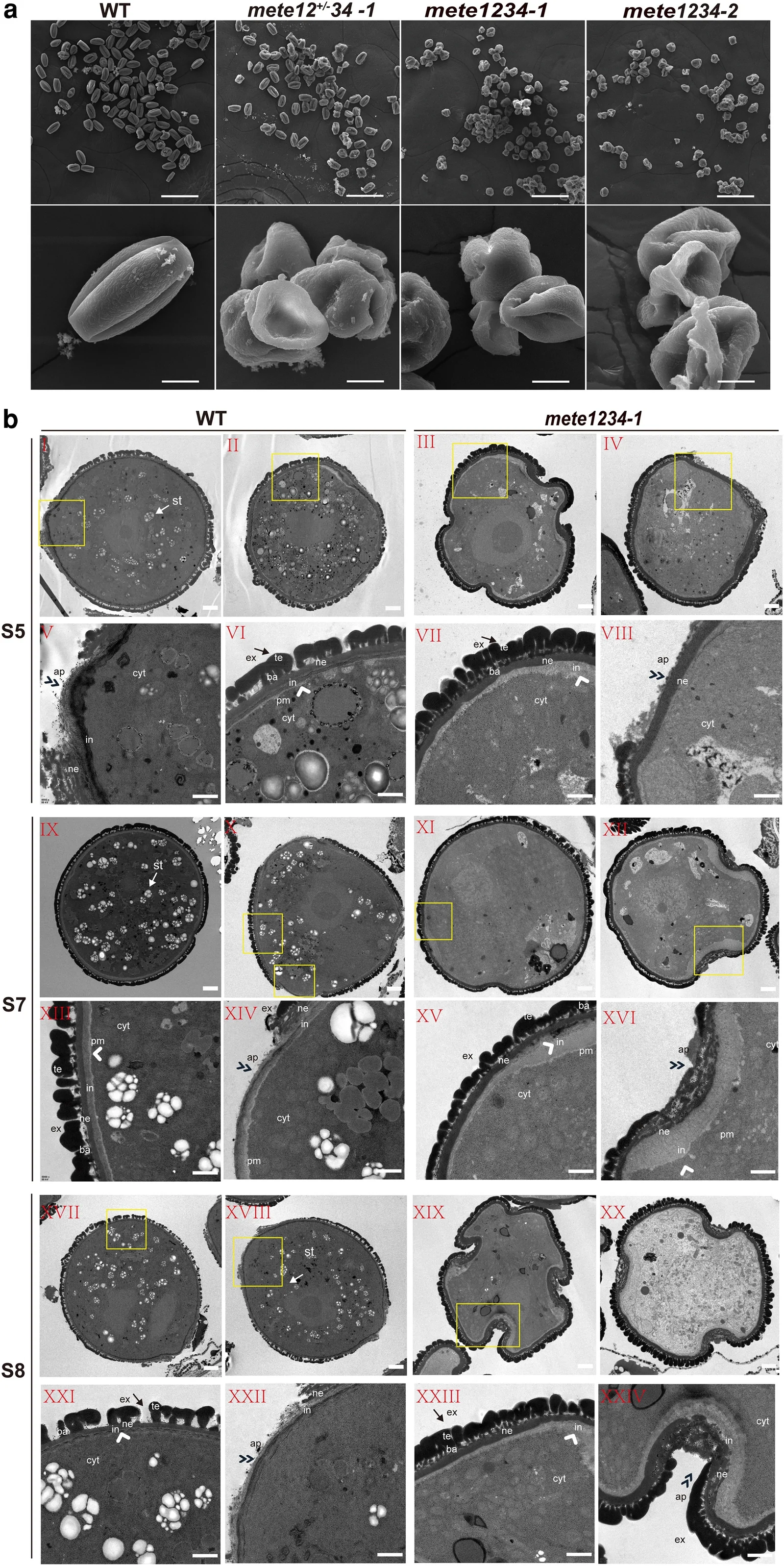
Knockout of *NtMetE*s impaired pollen wall and amyloplasts deposition. a) Scanning electron microscopy of pollen morphology in WT and *mete1234* mutants. Scale bars: top row, 100 μm; bottom row, 10 μm. b) Transmission electron microscopy observation of pollen ultrastructure in *mete1234*. White arrows in the first column indicate amyloplasts; white arrowheads indicate intine; black arrows mark the sporopollenin exine; double arrowheads indicate the aperture region. st, starch granule; ex, exine; in, intine; ne, nexine; ba, bacula; te, tectum; pm, plasma membrane; cyt, cytoplasm. Scale bars of the left 2 columns, 2 μm; right 4 columns, 1 μm. Images V–VIII represent magnified views of the regions of interest (ROIs) marked by yellow rectangles in images I–IV, respectively. Images XIII and XIV represent magnified views of the upper and lower yellow rectangles in image X, respectively, whereas images XV and XVI represent magnified views of the regions marked by yellow rectangles in images XI and XII, respectively. Images XXI and XXII represent magnified views of the regions marked by yellow rectangles in images XVII and XVIII, respectively, whereas images XXIII and XXIV represent magnified views of the regions marked by yellow rectangles in image XIX.

To explore why pollen viability and germination ratio decreased in the mutants, we further examined the pollen ultrastructure by TEM. The results revealed that pollen of both *mete12^+/−^34* and *mete1234* were shriveled (Fig. 6a). The percentage of shriveled pollen grains exceeded 45% in *mete1234*, whereas it was less than 7% in WT (Fig. 5h). In addition, in vitro germination and microscopic observation showed that there were normal-sized germinated grains (16.69%), normal-sized but ungerminated grains (26.03%), and smaller ungerminated grains in the mutants (57.28%) (Figure S6). These results indicated that *NtMetE*s were essential for maintaining pollen morphology and size.

As pollen shriveled after the binucleate stage, when the pollen wall develops, and amyloplasts deposit (Liao et al. 2020a, 2020b). We further compared the pollen ultrastructure between *mete1234* and WT at stages 5, 7, and 8. The results revealed that amyloplasts were deposited from stage 5, and became abundant at stages 7 and 8 in WT pollen (Fig. 6b). However, few amyloplasts were deposited in the pollen of *mete1234* at stages 5, 7 and 8 (Fig. 6b), suggesting that *NtMetE*s play a crucial role in regulating amyloplasts deposition during pollen maturation.

In addition, sporopollenin was absent in the aperture region of WT pollen but was present in *mete1234* pollen (Fig. 6b). The tectum and baculum of the pollen exine were more densely deposited in the nonaperture area, and the intine at the aperture area was thicker in the pollen of *mete1234* (Fig. 6b). These data reveal that *NtMetE*s also affect pollen wall development.

### Starch and sucrose, and citrate cycle metabolism, were impaired in the pollen of *mete1234*

**Figure 7:**
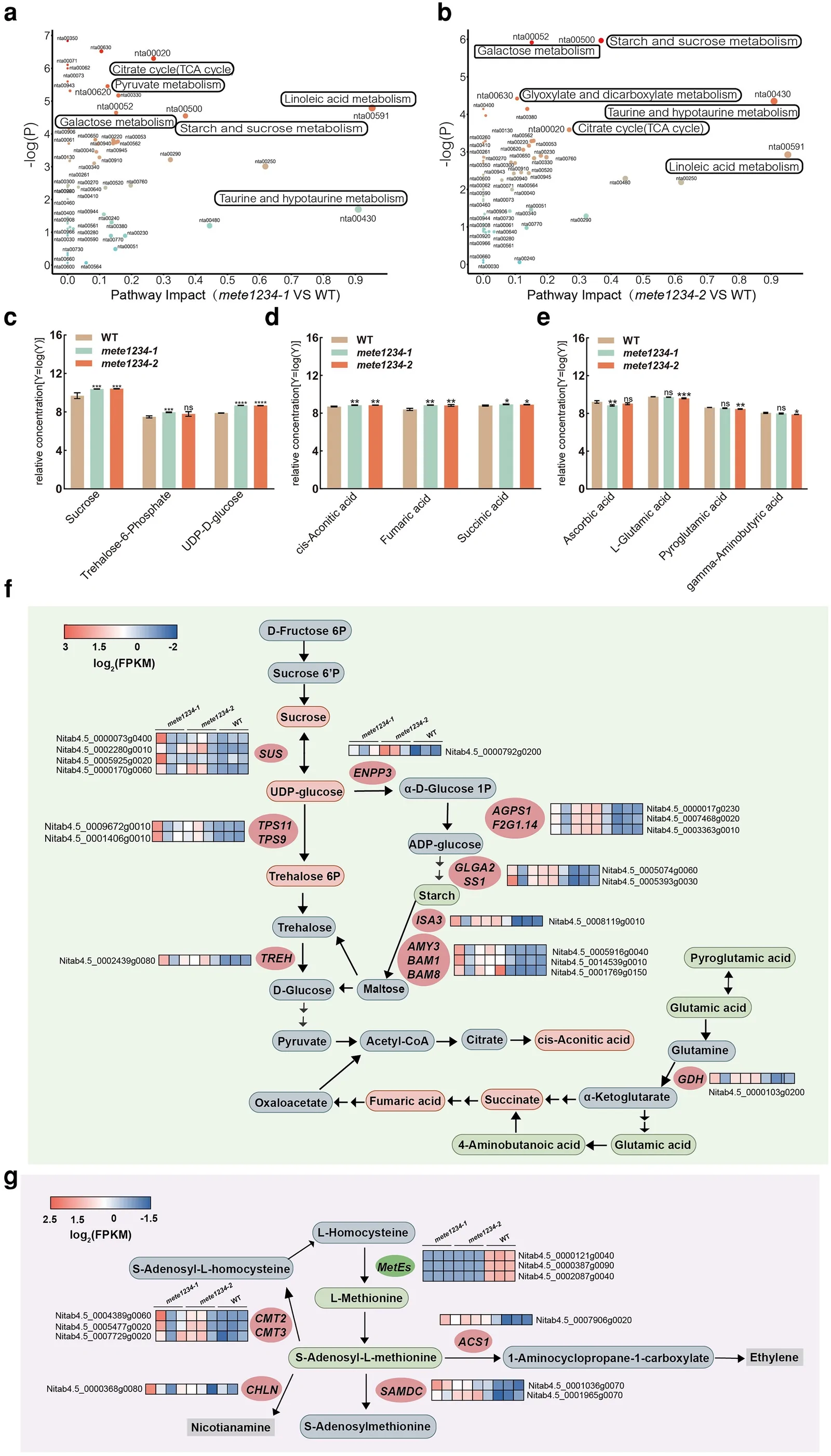
Changes in carbohydrate metabolism and gene expression in *mete1234*. a, b) KEGG enrichment analysis of differential metabolites between pollen of WT and *mete1234*. c) Relative levels of differential metabolites in sucrose and starch metabolism pathway. d) Relative levels of differential metabolites in the citrate (TCA) cycle. e) Relative levels of differential metabolites in the glutathione metabolism pathway. Data in (c–e) are means ± SD from independent biological replicates: WT (*n* = 3), *mete1234-1* (*n* = 4), *mete1234-2* (*n* = 4). f) Changes in metabolite abundance and gene expression related to starch, sucrose, and TCA cycle pathways. *SUS*, sucrose synthase; *ENPP3*, ectonucleotide pyrophosphatase/phosphodiesterase; *TPS9/11*, trehalose-6-phosphate synthases; *TREH*, trehalase; *AGPS1*/*F2G1.14*, glucose-1-phosphate adenylyltransferase small/large subunits; *GLGA2/SS1*, soluble starch synthases; *ISA3*, isoamylase 1; *AMY3*, alpha-amylase; *BAM1/8*, beta-amylases; *GDH2*, glutamate dehydrogenase. g) Changes in metabolite abundance and gene expression related to Met and SAM metabolism. *MetE*s, 5-methyltetrahydropteroyltriglutamate–homocysteine methyltransferase; *CMT2/3*, DNA (cytosine-5)-methyltransferase; *CHLN*, nicotianamine synthase; *ACS1*, ACC synthase; *SAMDC*, SAM decarboxylase. Uppercase letters in coral background represent upregulated genes. In f and g, light coral background represents the increased metabolites, while light olive background represents the metabolites with decreased content. Heat map represents the gene expression patterns. Statistical analysis was performed using one-way ANOVA (ns, nonsignificant; *P < 0.05; **P < 0.01; ***P < 0.001; ****P < 0.0001). Error bars represent SD from 3 technical replicates.

To investigate how *NtMetE*s influence amyloplast deposition in pollen, we performed transcriptomic and metabolomic analysis (Tables S5 and S6). The differential metabolites were enriched predominantly in pathways such as linoleic acid metabolism (nta00591), starch and sucrose metabolism (nta00500), citrate cycle (tricarboxylic acid [TCA] cycle, nta00020), glutathione metabolism (nta00480), and galactose metabolism (nta00052) (Fig. 7a and b). These pathways are related to carbohydrate and energy metabolism, and likely affect pollen maturation and fertility (Zhu et al. 2020; Lee et al. 2022).

We further identified the differential metabolites in these pathways. The results indicated that the contents of sucrose, trehalose 6-phosphate, and UDP-D-glucose from the starch and sucrose metabolism pathways were significantly elevated in *mete1234* pollen, with increased folds shown in Fig. 7c. Additionally, the levels of ascorbic acid, *cis*-aconitic acid, fumaric acid, and succinic acid of the TCA cycle also increased in *mete1234* pollen (Fig. 7d). In contrast, the levels of pyroglutamic acid, L-glutamic acid, and gamma-aminobutyric acid (which potentially enter the TCA cycle) significantly decreased (Fig. 7e). This reduction suggested that the elevated levels of TCA cycle metabolites did not result from increased flux through these upstream intermediates.

To explore how starch, sucrose, and energy metabolism are transcriptionally regulated in *mete1234* pollen, we further performed transcriptomic analysis. Kyoto Encyclopedia of Genes and Genomes (KEGG) analysis revealed that the differentially expressed genes (DEGs) were predominantly enriched in carbohydrate and lipid metabolism pathways (Figure S7a). The upregulated DEGs were enriched in galactose metabolism, fructose and mannose, and starch and sucrose metabolism pathways (Figure S7b and c). Specifically, upregulated DEGs of the starch and sucrose metabolism pathways included genes involved in the conversion of sucrose to D-glucose (such as sucrose synthase, trehalose-6-phosphate synthase, and trehalase), the catalysis of starch formation (eg ectonucleotide pyrophosphatase, glucose-1-phosphate adenylyltransferase, and starch synthase), and the degradation of starch (alpha/beta-amylase, isoamylase) (Fig. 7f). The upregulation of these genes and their associated metabolites, alongside reduced starch levels, may facilitate carbohydrate entry into the TCA cycle.

We further examined the expression of genes involved in Met and Sam metabolism in *mete1234* pollen. The transcript levels of *NtMetE1*, *NtMetE2*, and *NtMetE3* reduced by more than 40-fold in *mete1234* pollen, while the expression levels of genes catalyzing the transformation or recycling of Met, including those of *CHLN* (nicotianamine synthase), *CMT2/3* (DNA (cytosine-5)-methyltransferase CMT2/3), *ACS1* (aminocyclopropane-1-carboxylate synthase), and *SAMDC* (S-adenosylmethionine decarboxylase proenzyme), were upregulated (Fig. 7g**)**. This upregulation may be a compensatory response to reduced *NtMetE*s expression and diminished Met and Sam levels.

### *NtMetE*s-mediated changes in Met and Sam levels regulate anther/pollen development and pollen germination

**Figure 8:**
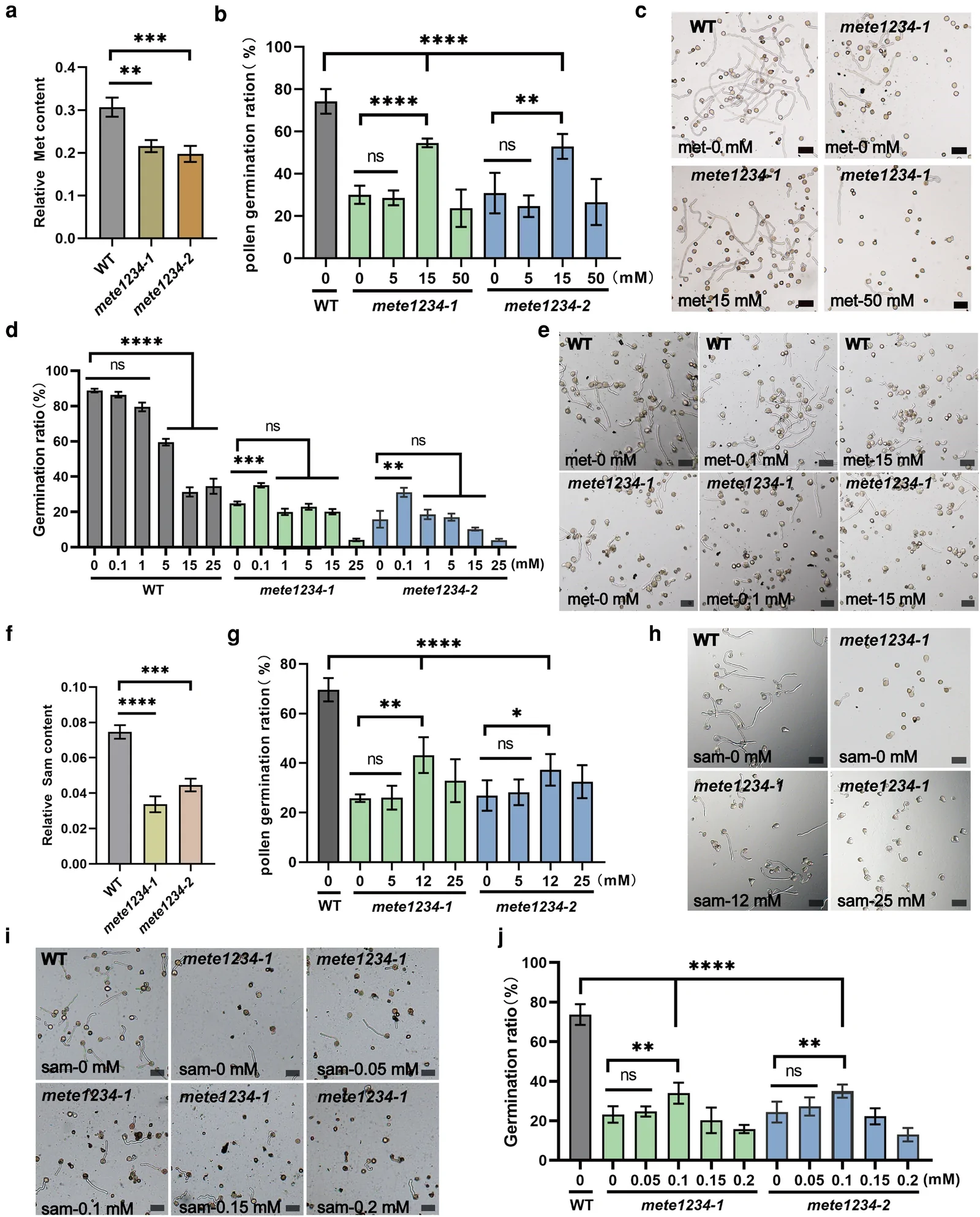
Met and Sam partially restored pollen germination of *mete1234*. a) The relative Met levels in the pollen of WT and *mete1234*; b, c) Application of Met on the flower bud of *mete1234* significantly rescues the pollen germination ratio. d, e) Application of Met on in vitro germinated pollen of *mete1234* significantly rescues the pollen germination ratio. f) The relative Sam levels in the pollen of WT and *mete1234*; g, h) Application of Sam on the flower bud of *mete1234* significantly rescues the pollen germination ratio. i, j) Application of Sam on in vitro–germinated pollen of *mete1234* significantly rescues the pollen germination ratio. For all plots, statistical analysis is conducted with ANOVA. Stars above the error bars represent statistical significance between each other. Bars = 100 μm. Each Met and Sam concentration was applied to 3 plants, and germination was tested in 3 replicates per plant (*n* = 600 pollen grains per replication). Statistical analysis was performed using one-way ANOVA (ns, nonsignificant; *P < 0.05; **P < 0.01; ***P < 0.001; ****P < 0.0001). Error bars represent SD. Scale bars = 100 μm.

NtMetEs function as key enzymes that methylate Hcy to Met, a precursor of Sam (Xia et al. 2024). The functional loss of *NtMetE*s might reduce the Met and Sam contents, and we therefore investigated whether NtMetEs regulate pollen germination through alterations in Met and Sam levels. LC-MS analysis revealed that the content of Met decreased by 29.41% to 5.68% in *mete1234* pollen (Fig. 8a). To assess whether exogenous Met could rescue pollen germination, we applied Met to in vitro cultured pollen (at stage 8). Treatment with 0.1 mM Met elevated the pollen germination ratio by an average of 12.78% (Fig. 8d and e) without changing the proportion of smaller-sized pollen (Figure S6f), while it reduced the percentage of normal-sized but ungerminated grains by 12.49%. Thus, Met directly rescues the germination of normal-sized pollen.

To explore if exogenous Met could affect anther and pollen development, we also applied Met to the flower bud of *mete1234* at stages 1 to 3. This application significantly increased the anther width, and rescued the pollen germination ratio by 22% to 25% (Fig. 8b and c, Figure S6a and b). Application of Met to flower buds reduced the percentage of normal-sized but ungerminated grains by 18.37%, while it only reduced the proportion of smaller-sized pollen by 11.09% (insignificant with that of untreated *mete1234*, Figure S6e and f). This implies that Met rescues pollen germination mainly by improving pollen germination capacity rather than by altering microspore development.

LC-MS analysis revealed that Sam levels were also reduced by 40.21% to 54.95% in *mete1234* pollen (Fig. 8f). To explore whether Sam reduction influences pollen germination, we applied exogenous Sam to in vitro cultured pollen. The results indicated that 0.1 mM of Sam increased the pollen germination ratio of *mete1234* by 10% (Fig. 8i and j), revealing that Sam can directly regulate pollen germination like that of Met. In addition, the application of 12 mM of Sam to the flower bud at stages 1 to 3 increased the pollen germination ratio of *mete1234* by 10% to 17% (Fig. 8g and h), revealing Sam also regulates pollen germination. These results collectively indicate that *NtMetE*s regulate anther development and pollen germination through Met and Sam. The rescue effect of Met is stronger than that of Sam, revealing that Met regulates pollen germination partially through the downstream Sam.

## Discussion

Normal pollen development and germination are crucial for the plant reproduction, breeding, and related industries (Althiab-Almasaud et al. 2024). *MetE*s are abundantly expressed in pollen, pollen tubes, and during pollen–pistil interactions (Kurvari et al. 1995; Endo et al. 2002; Moscatelli et al. 2005). However, their functions and regulatory mechanisms in pollen development and reproduction are still unknown, and the subcellular localization of MetEs requires further verification. In this study, we found that *NtMetE1-4*, abundantly expressed in anther and maturing pollen, redundantly regulate anther/pollen development and pollen germination. Functional loss of *NtMetEs* triggers 2 discrete, separable defects: (1) a severe developmental failure that generates a large fraction of morphologically aborted pollen, and (2) a functional impairment that prevents morphologically normal pollen from germinating. Functional impairment could be rescued by Met and Sam levels. Knockout of *NtMetE*s impaired carbohydrate, starch and sucrose, and TCA metabolism, reduced amyloplast deposition, and thickened intine in pollen. Furthermore, NtMetE1-6 belonging to the DS2 clade localized in the ER, cytosol, and nucleus, and the N-terminal transit peptide of NsylMetE4 affected its nucleus localization, revealing that Met synthesis and metabolism occurred widely in nucleus, cytosol, and ER.

### Met synthesis and metabolism genes exhibit diverse subcellular localizations

The MetEs of dicots can be classified into dicot subclade 1 (DS1) and dicot subclade 2 (DS2) (Silva Rody and de Oliveira 2018). DS2 members frequently lack transit peptides and are predicted to localize in the cytosol (Silva Rody and de Oliveira 2018). AtMetE1 and AtMetE2 (without the transit peptide, DS2 clade) are localized in the cytosol, and are primarily involved in the regeneration of Met (Ravanel et al. 2004; Silva Rody and de Oliveira 2018). Our data revealed that 6 NtMetEs of clade 2 do not contain the transit peptide, and these MetEs corresponded to those in the DS2 clade. These NtMetEs were localized in the ER, cytosol, and nucleus. The nuclear localization of MetEs is reported in *Pichia pastoris* and *Candida albicans* (Sahu et al. 2017), while the ER and nucleus localization of plant MetEs was also found.

Members of DS1 usually contain a transit peptide and are likely targeted to chloroplast (Silva Rody and de Oliveira 2018), and AtMetE3 of the DS1 clade (with the N-terminal transit peptide) is localized in plastids, and may be responsible for de novo Met synthesis (Ravanel et al. 2004). NsylMetE4 and NtomMetE4, with transit peptides, corresponded to proteins of the DS1 clade. NsylMetE4 was targeted to the nucleus and cytosol, and deletion of the transit peptide of NsylMetE4 weakened its nuclear localization. This localization is inconsistent with the prediction and the localization of AtMetE3 (Ravanel et al. 2004), revealing that this transit peptide does not necessarily determine chloroplast localization as reported (Silva Rody and de Oliveira 2018). Furthermore, as these sequences are not conserved among species, the subcellular localization it determines may also be diverse. Therefore, it is unreliable to infer the cytosol or plastid localization of MetEs according to their classification into the DS1 or DS2 clade.

The absence of plastid-localized MetEs in *Nicotiana* was consistent with the fact that they were also absent from some flowering plants, such as *Citrus sinensis*, *Lotus japonicus*, most monocots, and *Physcomitrium patens* (Silva Rody and de Oliveira 2018), and few MetEs maintain their original function as chloroplastic paralogs (Silva Rody and de Oliveira 2018). These data collectively indicate that chloroplast-localized MetEs are not indispensable for plant development.

Our data collectively reveal that the synthesis of Hcy occurred in the chloroplast, while the formation of Met may occur in the cytosol, ER, and nucleus, and its downstream metabolism and recycling occur widely in the cell membrane, cytosol, and nucleus. These findings indicate that Met and Sam are partitioned not only between the cytosol and organelles (Ravanel et al. 2004), but widely among the cell membrane, cytosol, organelles, and nucleus. Met plays a fundamental role in protein synthesis and maintains cellular redox balance, and also widely affects the Sam-mediated methylation reactions (occurring in various cellular compartments) polyamine and ethylene synthesis, etc. (Rudenko et al. 2024). The multicompartmentalization of Met synthesis and metabolism genes represents a fundamental biological strategy that ensures metabolic efficiency, enables specialized organelle functions, maintains cellular homeostasis, and reflects profound evolutionary adaptations (Sekula et al. 2020). These results improve our understanding of methionine synthesis, metabolism, and recycling in plants.

### *MetE*s of the DS2 clade are the major functional members involved in plant reproduction

*MetE*s are widely expressed during gamete adhesion, fertilization, anther development, and pollen tube growth, indicating their important role in plant reproduction (Kurvari et al. 1995; Endo et al. 2002; Moscatelli et al. 2005, 2015). However, there is currently no direct genetic evidence proving the function of *MetE*s in these processes. Our data provides genetic evidence indicating that *MetE*s play a vital role in anther and pollen development, pollen germination, and fruit set. *NtMetE1-4*, classified into clade 2 (DS2, without transit peptides), redundantly regulate anther/pollen development and pollen germination, while *NsylMetE4* and *NtomMetE4* in the ancestor genome (DS1, with transit peptide) might not be indispensable for plant reproduction and development, as their corresponding members were lost in allotetraploid offspring (*N. tabacum)*.

This inference is consistent with that AtMetE3 with the transit peptide exhibits very low expression levels and enzyme activities (Ravanel et al. 2004; Yan et al. 2019). Additionally, *AtMetE1* plays a predominant role in DNA and histone methylation among 3 *AtMetEs* homologs, and the homozygous *AtMetE1* mutation is lethal, whereas *AtMetE2* and *AtMetE3* may not play essential roles in chromatin silencing (Yan et al. 2019). These results indicate that *AtMetE1* is the major-effect gene, and that the functions of the 3 *AtMetE*s differ. Both the research on *Arabidopsis* and our data reveal that the MetEs of the DS2 clade are the major functional members involved in plant reproduction. Owing to their functional importance, the mutation of *MetEs* of DS2 clade was either lethal (Yan et al. 2019) or led to severe pollen deformity. This might be a protective mechanism to avoid the transmission of harmful mutations to offspring, which is essential for maintaining population health and stability.

### NtMetEs regulate anther/pollen development and pollen germination

Supplying Met/Sam to the medium partially restored the germination rate, reduced the percentage of normal-sized but ungerminated pollen, and did not alter the percentage of smaller-sized pollen, indicating a direct effect on pollen germination, providing compelling evidence that Met acts directly on pollen germination. Notably, rescue of pollen germination occurred only at specific concentrations of Met and Sam, rather than in a dose-dependent manner. This pattern indicates that excessive accumulation of Met or Sam might disrupt metabolic homeostasis or cause toxicity, consistent with the previous finding that elevated methionine levels can impair pollen tube growth (Chen et al. 2016).

During pollen germination, the stored carbohydrates, lipids, and proteins may be mobilized to meet the high energy demands (Rounds and Bezanilla 2013). The pollen of *NtMetE*s mutants accumulated less amyloplasts. This might restrict the energy supply during pollen germination. The supplied Met can be metabolized to enter central carbohydrate metabolism (eg TCA cycle) or glycolysis pathways, providing ATP and reducing cofactors (eg NADH, FADH2) (Rounds and Bezanilla 2013). In addition, methionine can be converted into 2-oxobutyrate. This acid is then further degraded into succinyl-CoA, a key substrate in cellular energy metabolism (Hildebrandt et al. 2015). These might explain how Met applied to the in vitro medium rescue pollen germination. *MAT3*-mediated Sam levels are essential for pollen germination and tube growth in *Arabidopsis thaliana* (Chen et al. 2016), consistent with that Sam levels reduced in *mete1234*, and the application of Sam could rescue pollen germination.

Met treatment of flower buds significantly increased anther width (Figure S6b and f), suggesting that exogenous Met improves sporophytic development. However, Met applied to the flower bud did not significantly reduce shriveled pollen ratio, suggesting that Met functions mainly by reactivating morphologically normal pollen that is metabolically arrested, rather than by affecting microspore development. These results are consistent with that *NtMetE*s are expressed mainly during pollen maturation (stages 5 to 8), and knockout of *NtMetE1-4* led to shrunken pollen at these stages, indicating that NtMetEs might function during pollen maturation rather than in early microsporogenesis or microspore development. Bud-applied Met could only reduce limited percentage of smaller-sized pollen; a plausible explanation is that reduced pollen size or structural collapse involves complex regulation of gene expression and metabolic pathways (Zhang et al. 2008). Exogenously supplied Met may be unable to penetrate these intricate biological processes and thereby alter the developmental fate of pollen exhibiting abnormal size or structure. This is consistent with that knockout of *NtMetE*s leads to severe gene expression and metabolism abnormality. The mechanisms by which Met and Sam modulate pollen maturation, morphological and structural development needs further verification.

### *NtMetE*s modulate energy metabolism and starch deposition

Carbohydrates have different fates: they are consumed immediately, transformed into other molecules, polymerized to form the intine, or stored as starch grains in amyloplasts (Pacini et al. 2006). Carbohydrate and energy metabolism disorders were detected in the pollen of *mete1234*. The DEGs and differential metabolites were enriched in carbohydrates, sucrose and starch metabolism pathways, and the contents of sucrose and UDP-D-glucose increased significantly. Furthermore, ultrastructural observations revealed that the inner wall of the pollen thickened in the aperture region. The inner layer of the pollen wall contains cellulose (Ma et al. 2021), and its synthesis requires UDP-D-glucose (Lee et al. 2012; Yang et al. 2020). The increased UDP-D-glucose contents in *mete1234* pollen might be related to their thicker inner wall deposition. This thickened inner wall and sporopollenin deposition at the aperture were also responsible for the decreased pollen germination ratio in *mete1234*.

Furthermore, UDP-D-glucose can be converted into pyruvate, which enters the TCA cycle through citrate (Liu et al. 2020). As the contents of *cis*-aconitic acid, succinate, and fumaric acid also increased significantly in the pollen of *mete1234*, we accordingly deduced that the flow of UDP-D-glucose into the TCA cycle may increase. The increased starch degradation might also lead to upregulated flow of UDP-D-glucose into the TCA cycle (Lu et al. 2005). Therefore, carbohydrates in the pollen of *mete1234* tend to be consumed through the TCA cycle or polymerized to form the intine rather than being stored as starch grains in amyloplasts. The amyloplast is the primary storage carbohydrate in mature pollen grains in many crop plants, including tobacco (Baker and Baker 1979; Lee et al. 2022; Cui et al. 2023). Impaired amyloplasts deposition might inhibit pollen germination owing to a shortage of energy and building blocks necessary for pollen germination and tube growth. This aligns with reports that starch-derived sugars limit TCA cycle activity and ATP production (Wu et al. 2023), and starch deficiency causes male sterility in rice by limiting energy and substrates for pollen germination (Arshad et al. 2017; Lee et al. 2022; Hsieh et al. 2023).

In addition, a reduction in the number of amyloplasts might also be related to high percentages of small/shriveled pollen in *mete1234*, consistent with reports that altered amyloplast-localized proteins affect starch grain number and pollen morphology in rice (Matsushima et al. 2013). Small/shriveled pollen might also be due to abnormal exine and intine development. As defective exine/intine development can disrupt pollen's turgor support, causing shrinkage and morphological collapse (Wang and Dobritsa 2021).

In summary, we clarified the phylogeny and subcellular localization of NtMetEs, as well as their functional divergence in plant reproduction and development. These findings advance our understanding of *MetE*s evolution and functional divergence, which is of great significance for understanding the important and widely influential biological processes of Met synthesis and metabolism. Additionally, we found *MetE*s regulate anther/pollen development and pollen germination through Met/Sam levels, and also modulated carbohydrate metabolism and amyloplasts deposition. These findings extend our knowledge of the fundamental life processes of plant reproduction.

## Materials and methods

### Plant materials and growth conditions

Seeds of the allotetraploid cultivar tobacco (*N. tabacum*) L. “K326” (K326, 2*n* = 24II = 48TTSS, WT), *Nothocnide repanda* (NR), and *Nicotiana stocktonii* (NS) were provided by the Yunnan Academy of Tobacco Agricultural Sciences. Transgenic lines deficient in *NtMetE1-4* were generated using the CRISPR/Cas9 system. All species and transgenic plants were sown in humus and cultured in a climate-controlled room (28 °C,16-h light, 8-h dark) for approximately 7 to 8 weeks. Subsequently, the plants were transplanted into soil in a greenhouse with an average temperature of 25 °C under natural sunlight, watered twice a week, and fertilized monthly with a water-soluble fertilizer (N:P:K = 20:20:20 + 0.5% trace elements) (Demei, Sichuan, China).

### NtmAb screening

We previously generated the monoclonal antibody slides (NtmAbs, including 15,000 antibodies) against the reproductive tissues of different *Nicotiana* species, which provided a powerful tool to identify the anther, pollen, and pistil abundantly expressed proteins (Chen et al. 2020). To identify the anther abundantly expressed proteins, mature anthers of K326, NR and NS, and pistils at 10 h after pollination (10 HAP) were collected. Proteins from these anthers and pistils were extracted for NtmAbs screening and WB analysis. NtmAbs screening was performed as described in our previous research (Chen et al. 2020). Proteins (0.1 μg μl^−1^) were labelled with NHS-biotin (Molecular Probes, Carlsbad, California, United States). The microarray slides were washed twice, and then blocked overnight at 4 °C with 10% BSA in TBS containing 0.1% Tween-20 (T20). After 4 washes with TBS + 0.5% T20 followed by 4 washes with double-distilled H_2_O, the slides were scanned using a GenePix microarray scanner (Axon Instruments, Union City, California, United States). Cy3 fluorescence was excited at 532-nm laser and detected at 570 nm. Cy3 fluorescence intensity was quantified using the GenePix 6.0 program (Axon Instruments), and further analyzed with the limma package (http://bioconductor.org/).

### WB analysis

Proteins (20 μg) from each sample were separated by SDS-PAGE and then transferred to polyvinylidene fluoride membranes. The membranes were incubated with mouse monoclonal primary antibodies from the NtmAb library, followed by horseradish peroxidase (HRP)-conjugated anti-mouse IgG secondary antibodies. Western blot (WB) analysis was performed as previously described (Chen et al. 2020).

### Immunoprecipitation (IP) and liquid chromatography (LC)–tandem mass spectrometry (MS/MS)

Total protein extracted from anthers (1 mg) was precleared with protein G resin (Invitrogen, Carlsbad, California, United States). After centrifugation, the supernatant was incubated with CNBr-conjugated antibody at 4 °C overnight. The immunoprecipitated (IP) complex was eluted with 0.2 M glycine (pH 2.5) and subjected to SDS-PAGE, followed by WB analysis. The corresponding band with expected molecular weight (MW) on WB was excised for in-gel trypsin digestion and MS identification.

The trypsin-digested proteins were analyzed by a nanoAquity UPLC system (Waters Corporation, Milford, Massachusetts, United States). Tandem mass spectra were processed using PEAKS Studio version 8.0 (Bioinformatics Solutions Inc., Waterloo, Ontario, Canada). PEAKS database was set up to search the NCBI *N. tabacum* database. PEAKS Q was used for calculating peptide and protein abundance. Differentially expressed proteins (DEPs) were defined as those with fold changes greater than 2.0 and *P*-values less than 0.05, as determined by one-way ANOVA.

### Genome-wide characterization of MetEs

The genomic data of *N. tabacum*, *N. sylvestris*, and *N. tomentosoformis* were downloaded from the NCBI database (GCA_000715135.1). The raw MetE domain hidden Markov model (HMM) files (PF01717) were downloaded from the Pfam database (http://pfam.xfam.org/) and used to search the genomic data using HMMER with a threshold *E*-value of 1e-20 (version 3.0, http://hmmer.janelia.org/) (Finn et al. 2015). The candidate sequences were further validated by domain analysis in SMART (http://smart.embl-heidelberg.de/), and the identified genes were designated as *MetE*s. MetE sequences of *Arabidopsis*, *S. tuberosum*, *S. lycopersicum*, and *Klebsormidium nitens* were downloaded from the NCBI database.

### Multiple sequence alignment and phylogenetic analysis

Multiple sequence alignments for MetE proteins were performed using ClustalW in MEGA7.0 with default parameters (Kumar et al. 2016) and manually modified with GeneDoc (http://github.com/karlnicholas/GeneDoc) (File S1). A phylogenetic tree was constructed using MEGA 7.0 with the maximum likelihood (ML) method with 1,000 bootstrap replicates. The MetE protein from *K. nitens* (GAQ81359.1) was used as the outgroup.

### Subcellular localization analysis

The complete ORF sequences of *NtMetE*s were amplified by primers containing KpnI and BamHI cleavage sites (Table S7). The purified PCR products were digested with KpnI and BamHI and cloned into the pCAMBIA1300 vector to generate NtMetE-GFP fusion protein under the control of CaMV 35S promoter. For construction of the 35S::sper::mKate::RFP vector, the endoplasmic reticulum signal peptide (MKTNLFLFLFLIFSLLLSLSSAEF) was fused to the far-RFP mKate of pBWA(V)HS-GLosgfp driven by the CaMV 35S promoter (Mravec et al. 2009). Both NtMetE::GFP and 35S::sper::mKate::RFP were transformed into *Arabidopsis* protoplasts isolated from mesophyll (Yoo et al. 2007; Mravec et al. 2009; Zhao et al. 2024), and NtMetE1::GFP was also transformed into *Nicotiana benthamiana* leaves. The 35S::GFP construct was used as a control. GFP fluorescence was excited at 488 nm and observed using a confocal microscope (Nikon C2-ER, Tokyo, Japan) after 18 to 24 h of transformation. The RFP emission signal was detected between 587 and 610 nm.

### RNA extraction and RT-qPCR

Total RNA was extracted from anthers, unpollinated pistils, mature pollen, and pollinated pistils using the MiniBEST Plant RNA Extraction Kit (Takara, Dalian, China). Gene-specific primer pairs were designed using Primer Premier 5.0, and their specificity was confirmed using NCBI Primer BLAST (Table S8). Reverse transcription, RT-qPCR, and data analysis were performed as described (Liao et al. 2020a, 2020b). The ubiquitin (*NtUBQ*) was used as reference gene (Schmidt and Delaney 2010). Three technical replicates were performed for each of the 3 biological replicates.

### Immunofluorescence (IF) analysis

Anthers of the WT and transgenic lines at different developmental stages were collected, immediately fixed with 4% paraformaldehyde (Beyotime, Shanghai, China) prepared in 20 mM PBS (pH 7.4), and processed as previously described (Brennan et al. 2019). For observation of anther development, samples were stained with aniline blue (Solarbio, Beijing, China). Pollen sample preparation and IF analysis were performed as previously reported (Liao et al. 2020a, 2020b), using primary antibodies against NtMetEs (N21 and N49, diluted 1:500).

### Paraffin sectioning, scanning electron microscopy (SEM) and transmission electron microscopy (TEM) analyses

For anther development analysis via paraffin sectioning, anthers of the WT and *mete1234* (T3 generation) plants at stages 1, 3, 5, and 8 were fixed in FAA and processed as previously described (Liao et al. 2020a, 2020b). For scanning electron microscopy (SEM) analysis, mature pollen from the WT and transgenic lines was collected and applied onto carbon adhesive tabs. The Au/Pd sputter coating process and imaging were performed as previously described (Jacobowitz et al. 2019). The pollen ultrastructure was observed using transmission electron microscopy (TEM). Anthers of the WT and *mete1234* (T3 generation) plants at stages 5, 7, and 8 were fixed in 5% glutaraldehyde prepared in phosphate-buffered saline (PBS) at 4 °C overnight, and subsequently processed as previously described (Liao et al. 2020a, 2020b).

### CRISPR/Cas9 vector construction and plant transformation

Two sequences (Table S9) targeting *NtMetE1-4* were inserted into the CRISPR/Cas9 pBWA(V)-zmpl vector (Ma et al. 2015). All the recombinant vectors were introduced into *Agrobacterium tumefaciens* GV3101 by electroporation. Stable transgenic lines were produced by *Agrobacterium*-mediated transformation of K326 (Horsch et al. 1985). The CRISPR/Cas9-edited lines were identified by sequencing. All primers used are listed in Table S9.

### Pollen viability, in vitro germination, and in vivo pollination assays

Fluorescein diacetate and propidium iodide (FDA/PI) (Solarbio, Beijing, China) was used to assess pollen viability (Wei et al. 2023). FDA/PI staining and imaging were performed as previously described (Muhlemann et al. 2018; Wei et al. 2023). For in vitro germination, fresh pollen from the WT and transgenic lines (T3 generation) was dusted onto minimal medium (10% sucrose, 0.005% boric acid, 0.08% agarose) or medium supplemented with different concentrations of Met or Sam. The pollen was incubated at 28 °C for 3 h. Pollen grains with tubes exceeding the grain diameter were considered germinated. Three biological replicates were performed.

Pollen germination and fruit weight were analyzed by in vivo pollination. Flowers of the WT and *mete134* (T2 generation) were subjected to both self-pollination and reciprocal pollination. Pistils at 24 HAP and unpollinated pistils of the WT and *mete134* were collected for RNA extraction. The expression levels of *NtMetE1*, *NtMetE2*, and *NtMetE3* were measured by RT-qPCR. The pollinated pistils (at 10 HAP) were fixed in aniline blue fluorochrome (ABF, Biosupplies, Parkville, Australia), and the pollen germination ratio was determined as described in our previous research (Liao et al. 2017). The fruit weight was analyzed at 45 HAP.

### Transcriptomic analysis

Pollen of WT and *mete1234* (T3 generation) at stage 5 was collected, immediately frozen in liquid nitrogen and stored at −80 °C (Liao et al. 2020a, 2020b). RNA extraction, library preparation, and processing of RNA-Seq data were performed as previously described (Ma et al. 2020). Briefly, total RNA was extracted using the TRIzol reagent (Invitrogen, California, United States) according to the manufacturer’s protocol. The libraries were constructed using VAHTS Universal V6 RNA-seq Library Prep Kit according to the manufacturer's instructions. The transcriptome sequencing and analysis were conducted by OE Biotech Co., Ltd. (Shanghai, China). Libraries were sequenced on an Illumina NovaSeq 6000 platform and 150-bp paired-end reads were generated. Differential expression analysis was performed using the DESeq2. A *q*-value <0.05 and fold change >2 or fold change <0.5 were set as thresholds for significantly DEGs. Based on the hypergeometric distribution, Gene Ontology (GO), KEGG pathway enrichment analysis of DEGs was performed to identify significantly enriched terms using R (v 3.2.0), respectively.

### Metabolomic analysis

Pollen of WT and *mete1234* (T3 generation) at stage 6 were collected, frozen in liquid nitrogen, and stored at −80 °C. Then, gas chromatography–mass spectrometry, data processing and analysis, and metabolic profiling were performed as previously described (Tian et al. 2018). Samples were processed using L-2-chlorophenylalanine (0.06 mg/mL in methanol) as an internal standard. Quality control (QC) samples were prepared by mixing an aliquot of all samples to be a pooled sample. Metabolomic data analysis was performed by Shanghai Luming Biological Technology Co., Ltd. (Shanghai, China). Raw LC-MS data were processed by software Progenesis QI V2.3 (Nonlinear, Dynamics, Newcastle, United Kingdom). Orthogonal Partial Least-Squares-Discriminant Analysis (OPLS-DA) and Partial Least-Squares-Discriminant Analysis (PLS-DA) were utilized to distinguish the metabolites that differed between groups. Differential metabolites were selected on the basis of VIP values >1.0 and *P*-values <0.05, and subjected to KEGG pathway enrichment analysis.

### Exogenous application of Met and Sam in vitro and in vivo

Met at concentrations of 5, 15, and 50 mM (Solarbio, Beijing, China) was sprayed onto the flower buds (stages 1 to 3) of the WT and *mete1234* (T3 generation) plants and reapplied 5 days later. Mature pollen (at stage 8) was harvested from the treated plants for in vitro pollen germination assays. For the in vitro assay, pollen from untreated *mete1234* and WT plants was collected, and Met was added to the germination medium at concentrations of 0.1, 1, 5, 15, and 25 mM. S-adenosylmethionine (Sam; Solarbio, Beijing, China) was applied in vivo at the concentrations of 5, 12, and 25 mM, and in vitro at 0.05, 0.1, 0.15, and 0.2 mM. The application method for Sam was the same as that used for Met.

### Statistical analysis

Statistical analysis was performed in IBM SPSS 26 and GraphPad Prism 8.3.0. Student's *t*-test or one-way ANOVA was performed to detect statistical differences. The phylogenetic tree was annotated using iTOL (https://itol.embl.de/). Figures were processed using ImageJ, Adobe Photoshop 2020, and Adobe Illustrator 2023.

### Accession numbers

Sequence data from this article can be found in the GenBank/EMBL data libraries under accession numbers. Accession numbers for tobacco methionine synthase genes are provided in Table S2, and accession numbers for MetE proteins from other species used in phylogenetic analyses are indicated in Fig. 2a.

## Supplementary Material

kiag572_Supplementary_Data

## Data Availability

The data supporting the findings of this study are available from NCBI under the accession number PRJNA1210479 (https://www.ncbi.nlm.nih.gov/sra/PRJNA1210479) and will be released on August 1, 2025.
