# Supplementary material for: Pollen-expressed methionine synthases in Nicotiana tabacum regulate pollen development and germination
Source: Plant Physiol. 2026 Aug 4;201(4):kiag572. doi: 10.1093/plphys/kiag572 (PMC13524084; doi:10.1093/plphys/kiag572)
Supplement: Supplementary file 1 [file kiag572_supplementary_data.zip › Supplemental information.docx]

**Pollen-expressed MetEs in *Nicotiana tabacum* regulate pollen maturation and germination by promoting amyloplast deposition**

Jie Shu^1,5^, Han Hu^1,5^, Keliang Tao^1^, Cai Feng^1^, Zhuo Wei^1^, Jiayuan Chang^1^, Xiaojun Qin^1^, Gonglin Chen^1^, Han Wu^1^, Gang Deng^1^, Yongzhi Niu^3^, Mingqiao Wang^2^, Yunye Zheng^3*^, Suiyun Chen^1^*, Jugou Liao^1^*

^1^ School of Ecology and Environmental Sciences, Yunnan University, Biocontrol Engineering Research Center of Crop Diseases & Pests, Ministry of Education Key Laboratory for Transboundary Ecosecurity of Southwest China, Kunming, Yunnan, 650091, China

^2^ School of Life Sciences, Yunnan University, Yunnan Province, Kunming, 650091, China

^3^ Abmart, Shanghai, 200033, China

^4^ Yuxi Zhongyan Tobacco Seed CO., Ltd., Yuxi, Yunan, 653100, China

^5^ These authors contributed equally to this work.

*To whom correspondence should be addressed Z.Y (zyy@tobacco-seed.com), S.C (chensuiyun@ynu.edu.cn), or J.L. (liaojugoulove@126.com)

**Supplemental Table S1. Anther or pistil abundantly expressed proteins identified by IP-LC-MS/MS**

| CL number | Uniprot accession | Mw  (kD) | Protein name | Abbreviation |
| --- | --- | --- | --- | --- |
| N000009 | A0A1S4DH74 | 63 | beta-fructofuranosidase, insoluble isoenzyme CWINV1-like | Beta-FFase |
| N000010 | A0A1S3Z449 | 68 | beta-glucosidase BoGH3B-like | BoGH3B |
| N000011 | A0A1S4BXK3 | 53 | Endoglucanase | EG |
| N000031 | A0A1S3YQV4 | 23 | HMG-Y-related protein B-like | HMG-Y |
| N000032 | A0A1S3ZJV3 | 17 | class I heat shock protein-like | sHSP |
| N000034 | A0A1S3Z858 | 50 | apyrase-like | APY |
| N000036 | A0A1S4CZ78 | 40 | polygalacturonase inhibitor-like | PGIP |
| N000038 | A0A1S3YNC4 | 40 | Glutamine synthetase | GS |
| N000040 | A0A1S4B7N5 | 70 | polyphenol oxidase E chloroplastic-like | PPO |
| N000043 | A0A1S3YVF2 | 65 | polyphenol oxidase E chloroplastic | PPO |
| N000044 | A0A1S3Y633 | 23 | vicilin-like seed storage protein | SSP |
| N000047 | P68173 | 50 | Adenosylhomocysteinase | SAHH |
| N000049 | A0A1S3ZYM9 | 80 | 5-methyltetrahydropteroyltriglutamate- homocysteine methyltransferase-like | MetE |
| N000050 | A0A1S3ZAU1 | 22 | glycine-rich RNA-binding protein-like | GRP |
| N000051 | A0A1S4BDR4 | 50 | acetolactate synthase small subunit 1 chloroplastic like | ALS |
| T07 | Q9M7K8 | 48 | S-adenosylmethionine synthase 1 | SAMS1 |

**Supplemental Table S2. The basic information and physicochemical characteristics of NtMetEs**

| Gene ID | Protein ID | Length(aa) | Abbreviation |
| --- | --- | --- | --- |
| LOC107797732 | XP_016476141.1 | 765 | NtMetE1 |
| LOC107811094 | XP_016491448.1 | 765 | NtMetE2 |
| LOC107763602 | XP_016437579.1 | 765 | NtMetE3 |
| LOC107791827 | XP_016469454.1 | 765 | NtMetE4 |
| LOC107768359 | XP_016442967.1 | 765 | NtMetE5 |
| LOC107827442 | XP_016510071.1 | 765 | NtMetE6 |
| LOC104108327 | XP_009615630.1 | 765 | NtomMetE1 |
| LOC104119942 | XP_009629870.1 | 765 | NtomMetE2 |
| LOC104111436 | XP_009619432.1 | 765 | NtomMetE3 |
| LOC104088570 | XP_009591564.1 | 829 | NtomMetE4 |
| LOC104249971 | XP_009804811.1 | 765 | NsylMetE1 |
| LOC104237549 | XP_009790020.1 | 765 | NsylMetE2 |
| LOC104238725 | XP_009791486.1 | 765 | NsylMetE3 |
| LOC104224931 | XP_009774960.1 | 830 | NsylMetE4 |

**Supplemental Table S3. The sequence similarity of NtMetEs**

| Protein | NtMetE1 | NtMetE2 | NtMet3 | NtMetE4 | NtMetE5 | | NtMetE6 |
| --- | --- | --- | --- | --- | --- | --- | --- |
| NtMetE1 | 100.00 |  | | | | | |
| NtMetE2 | 98.82 | 100.00 |  | | | | |
| NtMetE3 | 95.82 | 95.16 | 100.00 |  |  | | |
| NtMetE4 | 96.21 | 95.56 | 98.43 | 100.00 | |  | |
| NtMetE5 | 95.95 | 95.56 | 96.34 | 95.95 | 100.00 | |  |
| NtMetE6 | 95.82 | 95.56 | 96.21 | 95.82 | 99.35 | | 100.00 |

**Supplemental Table S4. Primers used to detect the expression levels of *NtMetEs* by RT-qPCR**

| Primer name | Sequence（5'→3'） |
| --- | --- |
| *NtMetE1*-F | TTCTCTTTGCCCACGTTCATATAC |
| *NtMetE1*-R | ACTGCTCTTTCCATCCCAGAA |
| *NtMetE2*-F | GCCACGTTCAATATAGTTCTCTTT |
| *NtMetE2*-R | ATCCCAGAAAGATTCGAGAGCA |
| *NtMetE3*-F | TGTTGTTGTTTCTTCGCCAAT |
| *NtMetE3*-R | ACCTTCTTCAAGTCCTCAGCG |
| *NtMetE4*-F | TGGTCCTCAGCTGCTCAAAG |
| *NtMetE4*-R | AGGTCTCAAACCTTGGCTGG |
| *NtMetE5*-F | CTGAGGTAAAGCCAGCCCTC |
| *NtMetE5*-R | CCCACGCCTTCAATCTGACT |
| *NtMetE6*-F | GAAGGAGTCGCGACATATCA |
| *NtMetE6*-R | GGGCAGAGCTAACCTACACA |
| *Ntubc2*-F | CTGGACAGCAGACTGACATC |
| *Ntubc2*-R | CAGGATAATTTGCTGTAACAGATTA |

| Usage | Primer name | Sequence（5'→3'） |
| --- | --- | --- |
| Primers to  clone  *NtMetEs* into  pBWA(V)HS-  GLosgfp | *NtMetE1*-SF | CAGTCGTCTCA/CAACATGGCATCTCACATAGTTGG |
|  | *NtMetE1*-SR | CAGTCGTCTCA/TACACTTGGCGCTGGCAAGCTGGG |
|  | *NtMetE2*-SF | ATTTGGAGAGGACAGGGTACC/ATGGCATCTCACATAGTTGGAT |
|  | *NtMetE2*-SR | GGCAGCAGCTCTAGAGGATCC/AACCATGGCTTGGAGTGC |
|  | *NtMetE3-SF* | ATTTGGAGAGGACAGGGTACC/ATGGCATCTCACATTGTTGGAT |
|  | *NtMetE3-SR* | GGCAGCAGCTCTAGAGGATCC/CTTGGCACTGGCAAGCTGA |
|  | *NtMetE4-SF* | ATTTGGAGAGGACAGGGTACC/ATGGCATCTCACATTGTTGGATACC |
|  | *NtMetE4-SR* | GGCAGCAGCTCTAGAGGATCC/AACTCTATCGGCTATCTCCTCTGTG |
|  | *NtMetE5-SF* | CCTTCAGTTTCGCGAGCTCGGTACC/ATGGCATCTCACATTGTTGGATACC |
|  | *NtMetE5-SR* | TTGCTCACCATGGTTCTAGAGGATCC/AACTCTATCGGCTATCTCCTCTGTG |
|  | *NsylMetE4-Ndel-SF* | ATTTGGAGAGGACAGGGTACC/ATGGCATCTCACGTTGTTGGA |
|  | *NsylMetE4-Ndel-SR* | GGCAGCAGCTCTAGAGGATCC/CTTGGTGCTGGCCAAGTGT |
|  | *NtCBL-SF* | ATTTGGAGAGGACAGGGTACC/ATGTCATATTCTGTGTCTCTCAAGCAG |
|  | *NtCBL-SR* | GGCAGCAGCCTAGAGGATCC/TGCTGGCCCAGTCCTGA |
|  | *NtSAHH-SF* | ATTTGGAGAGGACAGGGTACC/ATGGCTCTATTAGTCGAGAAGACCA |
|  | *NtSAHH-SR* | GGCAGCAGCTCTAGAGGATCC/GTACCTGTAGTGAGCAGGCTTG |
|  | *NtSAM-SF* | ATTTGGAGAGGACAGGGTACC/ATGGAAACTTTCTTGTTCACCTCAGA |
|  | *NtSAM-SR* | GGCAGCAGCTCTAGAGGATCC/AGCTTTTGGCTTGAGGACCT |

**Supplemental Table S5. Primers used to explore the subcellular localization of *NtMetEs***

Note: Underlined nucleotides indicate enzyme cleavage sites.

**Supplemental Table S6. Primers and targets for constructing the *NtMetEs* mutants by CRISPR-Cas9**

|  | Primer name | Sequence（5'→3'） |
| --- | --- | --- |
| Targets (red)  and their  cloning  primers | sgRNA-1-F | CAGTGGTCTCA/TGCACGGTCCAGTTGTATCTAGAT |
|  | sgRNA-1-R | CAGTGGTCTCA/AAAC**ATCTAGATACAACTGGACCG** |
|  | sgRNA-2-F | CAGTGGTCTCATGCAGTCGAATCCGATCTCACCAC |
|  | sgRNA-2-R | CAGTGGTCTCAAAAC**GTGGTGAGATCGGATTCGAC** |
|  | M13-F | CCAGAAATTGAACGCCGAAG |
|  | zmpl-R | GTAAAACGACGGCCAGT |
| Primers for  mutants  identification | *NtMetE1*-MF | AAGAATGGATCTTTACTAGTATG |
|  | *NtMetE1*-MR | GGATCAAATAGTTATGATCAGTATG |
|  | *NtMetE2*-MF | TCTGGAGTTATAGATTTGATCG |
|  | *NtMetE2*-MR | ATCAAATAGTGATGATCAGTATG |
|  | *NtMetE3-MF* | CAAACGCGAGCTAAGTATT |
|  | *NtMetE3-MR* | GTATACATAAGAAAAGAATCACATA |
|  | *NtMetE4-MF* | GTGTGAATAAAGAATGAGTTTAAG |
|  | *NtMetE4-MR* | ATCAGTCTTTCTAGAGTCTAGA |

**Supplemental Table S7. The edited sequence of the mutants**

| Mutant | Sequence（5'→3'） | Type of mutation |
| --- | --- | --- |
| *mete23-1/2* | *NtMetE2：*CCGGTG TGAGATCGGATTCGAC | deletion of G base |
|  | *NtMetE3：*CCAGTGAGTGAGATCGGGTTCG | insertion of A base |
| *mete14-1/2* | *NtMetE1：*CCGGTGAGTGAGATCGGATTCGAC | insertion of A base |
|  | *NtMetE4：*CCGGTGAGTGAGATCGGGTTTGAC | insertion of A base |
| *mete124-1/2* | *NtMetE1：*CCGGTGAGTGAGATCGGATTCGAC | insertion of A base |
|  | *NtMetE2：*CCGGTGAGTGAGATCGGATTCGAC | insertion of A base |
|  | *NtMetE4：*CCGGTGAGTGAGATCGGGTTTGAC | insertion of A base |
| *mete134-1/2* | *NtMetE1：*CCGGTGAGTGAGATCGGATTCGAC | insertion of A base |
|  | *NtMetE3：*CCAGTGAGTGAGATCGGGTTCG | insertion of A base |
|  | *NtMetE4：*CCGGTGAGTGAGATCGGGTTTGAC | insertion of A base |
| *mete12^+-^34-1/2* | *NtMetE1：*CCGGTGAGTGAGATCGGATTCGAC | insertion of A base |
|  | *NtMetE2-1：*CCGGTGCGTGAGATCGGATTCGAC | insertion of C base |
|  | *NtMetE2-2：*CCGGTG AGATCGGATTCGAC | deletion of GTG base |
|  | *NtMetE3：*CCAGTGAGTGAGATCGGGTTCG | insertion of A base |
|  | *NtMetE4：*CCGGTGTGTGAGATCGGGTTTGAC | insertion of T base |
| *mete1234-1/2* | *NtMetE1:*CCGGTGAGTGAGATCGGATTCGAC | insertion of A base |
|  | *NtMetE2：*CCGGTGCGTGAGATCGGATTCGAC | insertion of C base |
|  | *NtMetE3：*CCAGTGAGTGAGATCGGGTTCG | insertion of A base |
|  | *NtMetE4-1：*CCGGTGTGTGAGATCGGGTTTGAC | insertion of T base |
|  | *NtMetE4-2：*CCGGTG TGAGATCGGGTTTGAC | deletion of G base |
|  |  |  |
| target1:CCCATCTAGATACAACTGGACCG | |  |
| target2:CCGGTGGTGAGATCGGATTCGAC | |  |
