# Supplementary material for: Pollen-expressed methionine synthases in Nicotiana tabacum regulate pollen development and germination
Source: Plant Physiol. 2026 Aug 4;201(4):kiag572. doi: 10.1093/plphys/kiag572 (PMC13524084; doi:10.1093/plphys/kiag572)
Supplement: Supplementary file 1 [file kiag572_supplementary_data.zip › Supplementary Data_7.29.pdf]

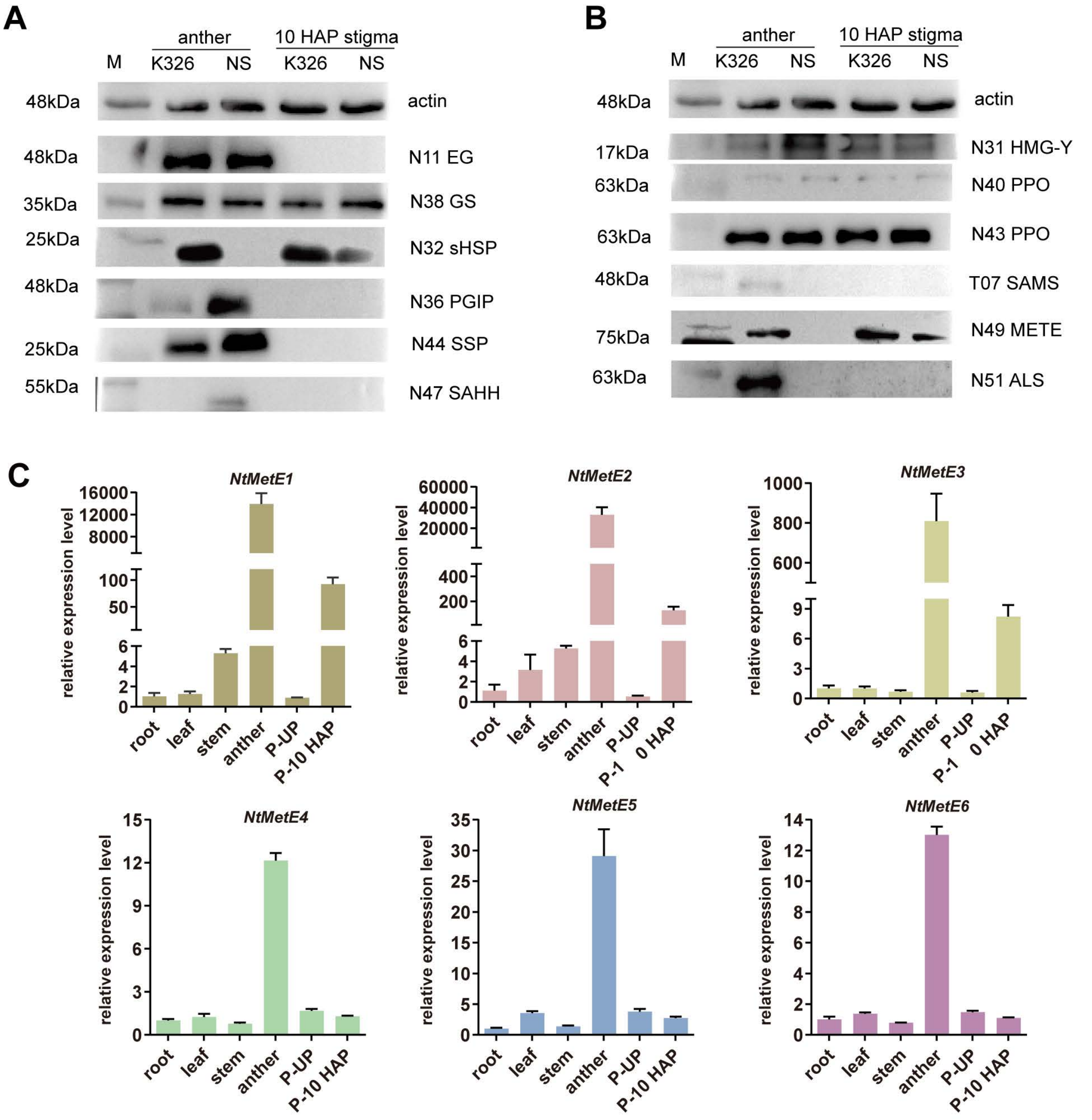

Supplementary Figure S1 WB analysis of DEPs identified by NtmAb screening and tissue expression analyses of *NtMetE*s

(A) Western blot (WB) analysis of differentially expressed proteins (DEPs) identified by NtmAb screening. (A) and (B) use the same actin protein blots as a control. K326 refers to *N. tabacum* L. “K326”; NS, *Nicotiana stocktonii*. “10 HAP” indicates 10 hours after pollination. Full protein names and molecular weights are listed in Supplementary Table 1. **(B)** Relative expression levels of *NtMetEs* detected by RT-qPCR. P, pistil, UP, unpollinated pistil; HAP, hours after pollination. Error bars represent the SD from three technical replicates. **(C)** RT–qPCR analysis of NtMetE1–6 expression in root, leaf, stem, anther, unpollinated pistil, and pistil at 10 days after pollination. Black lines indicate image boundary.

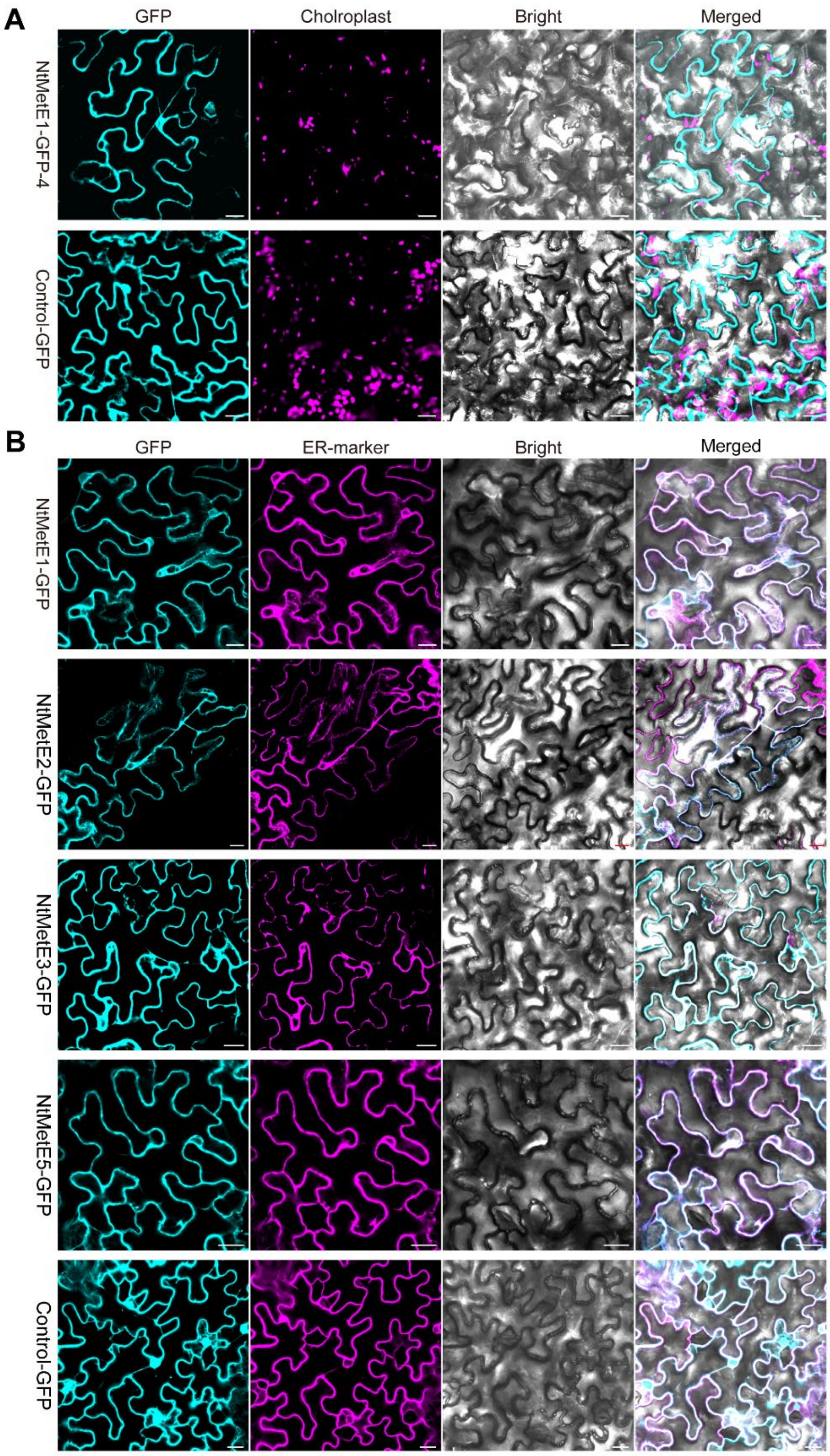

Supplementary Figure S2 Subcellular localization of NtMetEs in *Nicotiana benthamiana* leaves

Subcellular localization of NtMetE1, NtMetE2, NtMetE3, NtMetE5 and GFP by transient expression assays in *Nicotiana benthamiana* leaves. The ER marker (SPER-mKate) used in this study was adopted based on the reference (Zhao et al., 2024). Scale Bars = 10 μm.

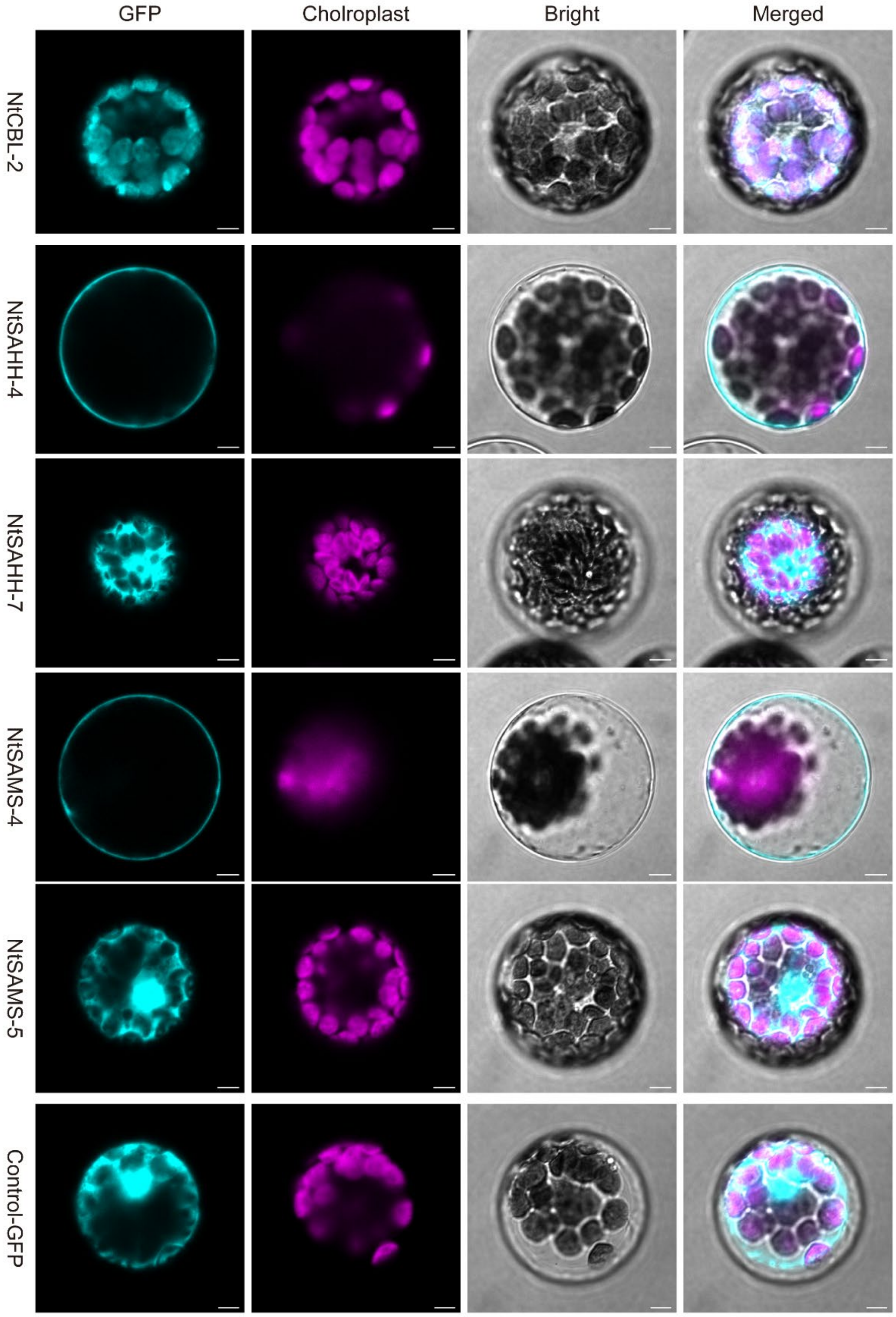

Supplementary Figure S3 Subcellular localization of NtMetE1 and NtCBL2, NtSAHHs, and NtSAMs

Subcellular localization of NtCBL2, NtSAHH4/7, NtSAM4/5 and GFP in *Arabidopsis thaliana* protoplasts. The ER marker (SPER-mKate) used in this study was adopted based on the reference (Zhao et al., 2024). Scale Bars = 10 μm.

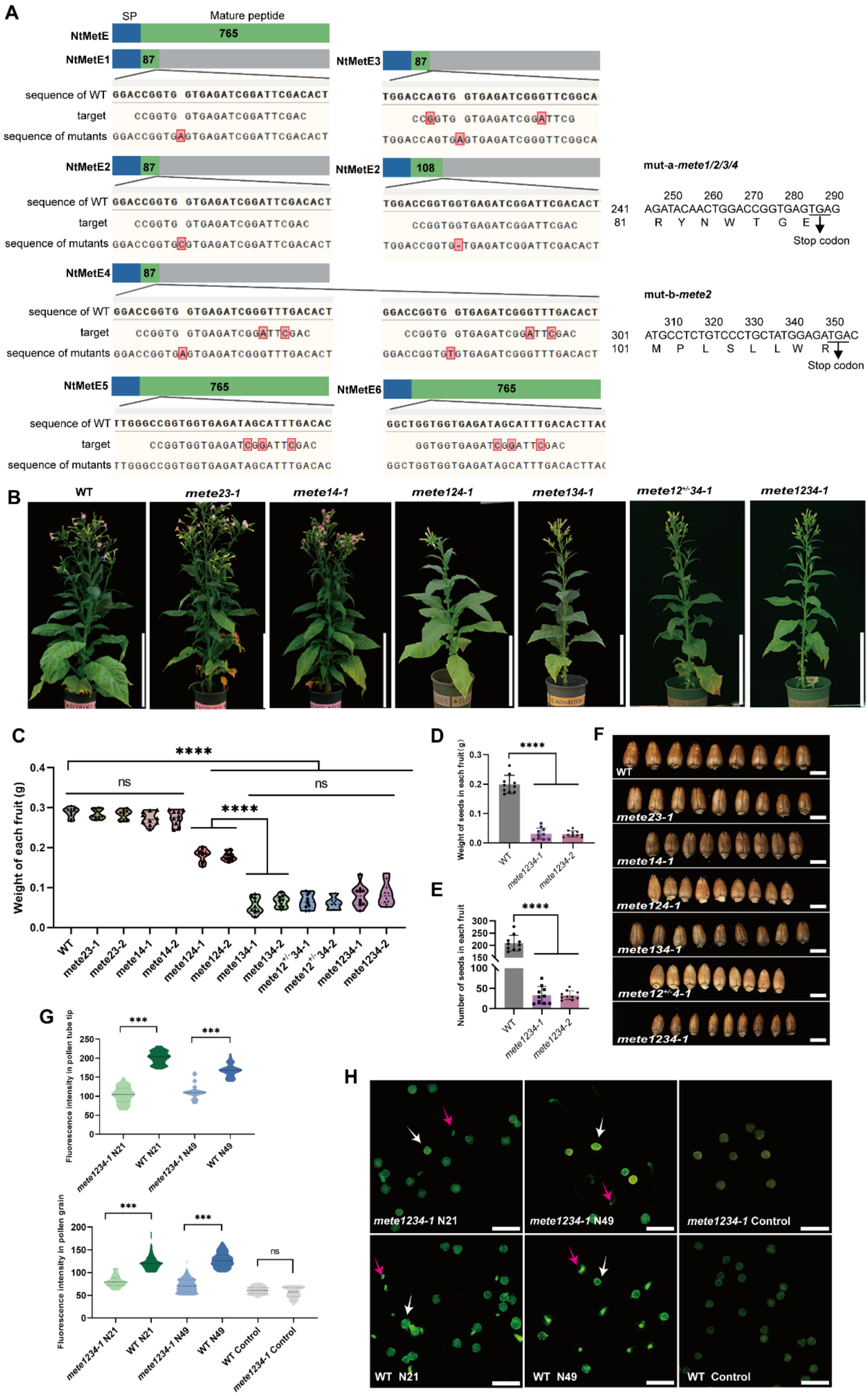

A
SP
Mature peptide
NtMetE
NtMetE1
NtMetE2
NtMetE3
NtMetE4
NtMetE5
NtMetE6
sequence of WT
target
sequence of mutants
mut-a-mete1/2/3/4
Stop codon
mut-b-mete2
B
WT
mete23-1
mete14-1
mete124-1
mete134-1
mete12+/-34-1
mete1234-1
C
Weight of each fruit (g)
D
Weight of seeds in each fruit (g)
E
Number of seeds in each fruit
F
G
Fluorescence intensity in pollen tube tip
Fluorescence intensity in pollen grain
H
mete1234-1 N21
mete1234-1 N49
mete1234-1 Control
WT N21
WT N49
WT Control

Supplementary Figure S4 Mutants identification, plant growth and fruit weight analysis

(A) CRISPR-Cas9 editing results of *NtMetE1-4*. Base insertions or deletions led to premature termination. *NtMetE5* and *NtMetE6* were not edited in any mutant lines. **(B)** Plant morphology of *mete13, mete14, mete124, mete134*, *mete12$^{+/-}$34*, and *mete1234*. Scale bar = 50 cm. **(C)** Fruit weight analysis of mutants (n = 10), Scale bar = 2 cm. **(D)** Seed weight in per fruit. Ten fruits were analyzed per mutant. **(E)** Seed number in per fruit. Ten fruits were analyzed per mutant plant. **(F)** Fruits of WT and mutants. **(G, H)** Immunofluorescence analysis of NtMetEs expression in pollen and pollen tubes in WT and *mete1234*. White arrows indicate pollen grains; red arrows indicate pollen tube tips. Scale bar = 50 μm. Quantification of green fluorescence intensity: n = 100 pollen grains, n = 30 pollen tube tips. Statistical analysis was performed using one-way ANOVA (ns, non-significant; *P < 0.05; **P < 0.01; ***P < 0.001; ***P < 0.0001). Error bars represent mean ± SD.

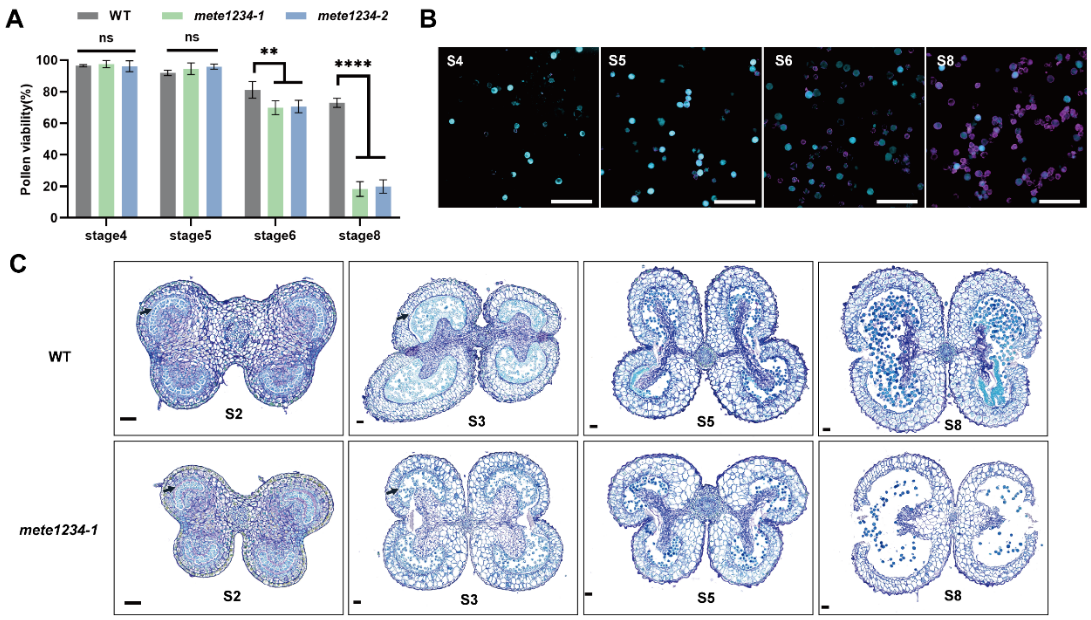

S Supplementary Figure S5 Effects of *NtMetEs* on pollen viability and anther structure

(A, B) Pollen viability analyzed by FDA-PI staining. Cyan indicates live pollen; magenta, dead pollen; Scale bar = 0.25 mm. **(C)** Anther structure analyzed by paraffin section. Arrows indicate the tapetum. Scale bar = 50 μm. All measurements were repeated three biological replicates. Statistical analysis was performed using one-way ANOVA (ns, non-significant; *$P < 0.05$; **$P < 0.01$; ***$P < 0.001$; ***$P < 0.0001$). Error bars represent SD.

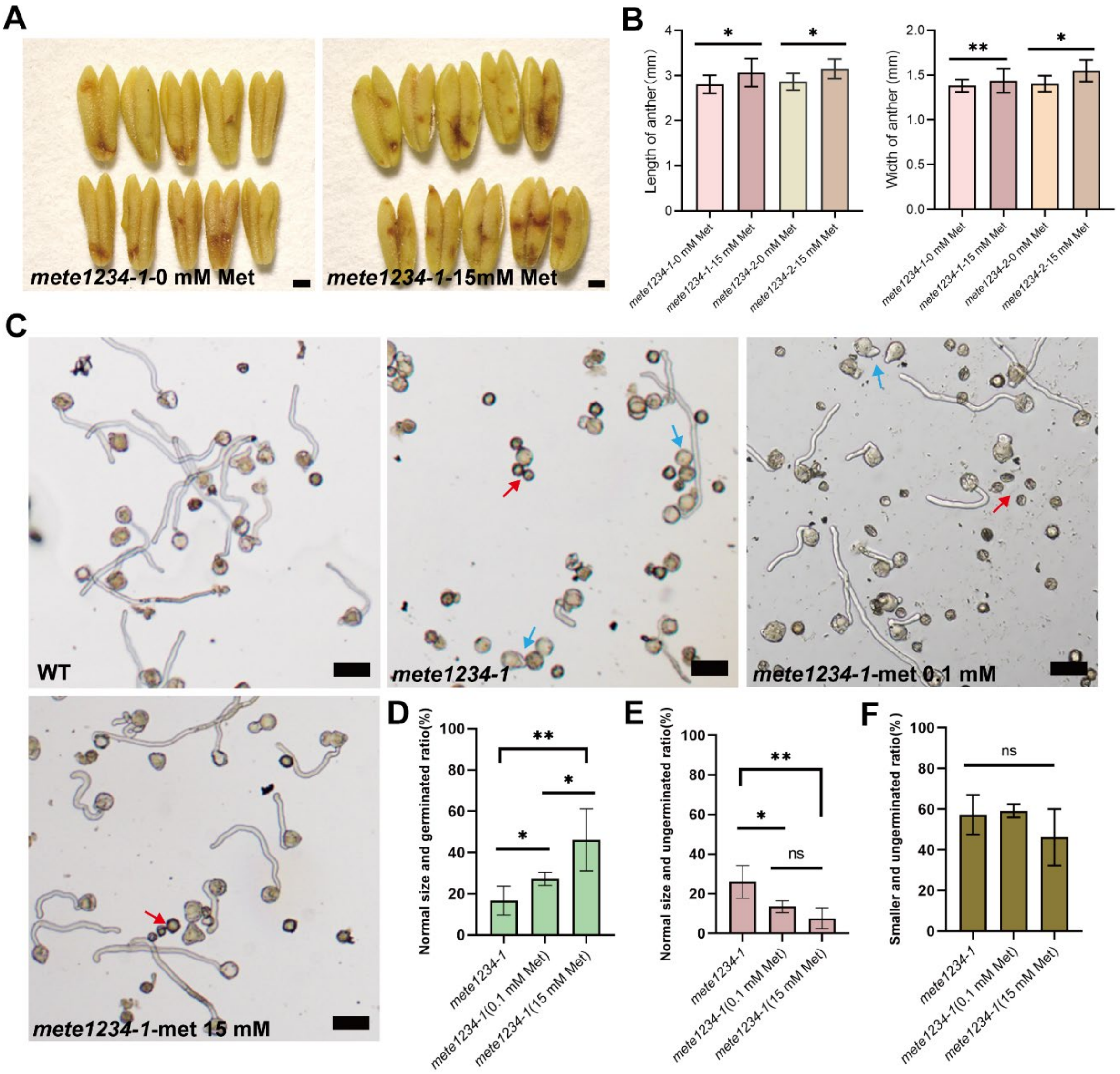

Supplementary Figure S6 Effect of Met on anther and pollen size

(A) Anthers morphology and size in *mete1234* sprayed with 15 mM of Met. Samples were photographed at the same time and are part of a single image (without rearrangement), bars = 0.5 mm, n =10 anthers. **(B)** Anther width and length in *mete1234* sprayed with 15 mM of Met. **(C and D)** The proportion of normal size and germinated pollen grains in *mete1234* before and after treatment with 0.1 mM of Met added *in vitro* germination medium, and 15 mM of Met applied to flower buds, bars = 50 μm. **(E)** The proportion of normal size and ungerminated pollen. **(F)** The proportion of smaller size and ungerminated pollen. Smaller pollen is defined as pollen that failed to germinate and has a diameter smaller than the average diameter of germinated pollen. Count approximately 30 pollen grains per field of view, n=5. Statistical analysis was performed using T-test (ns, non-significant; **P* < 0.05; ***P* < 0.01). Error bars represent SD.

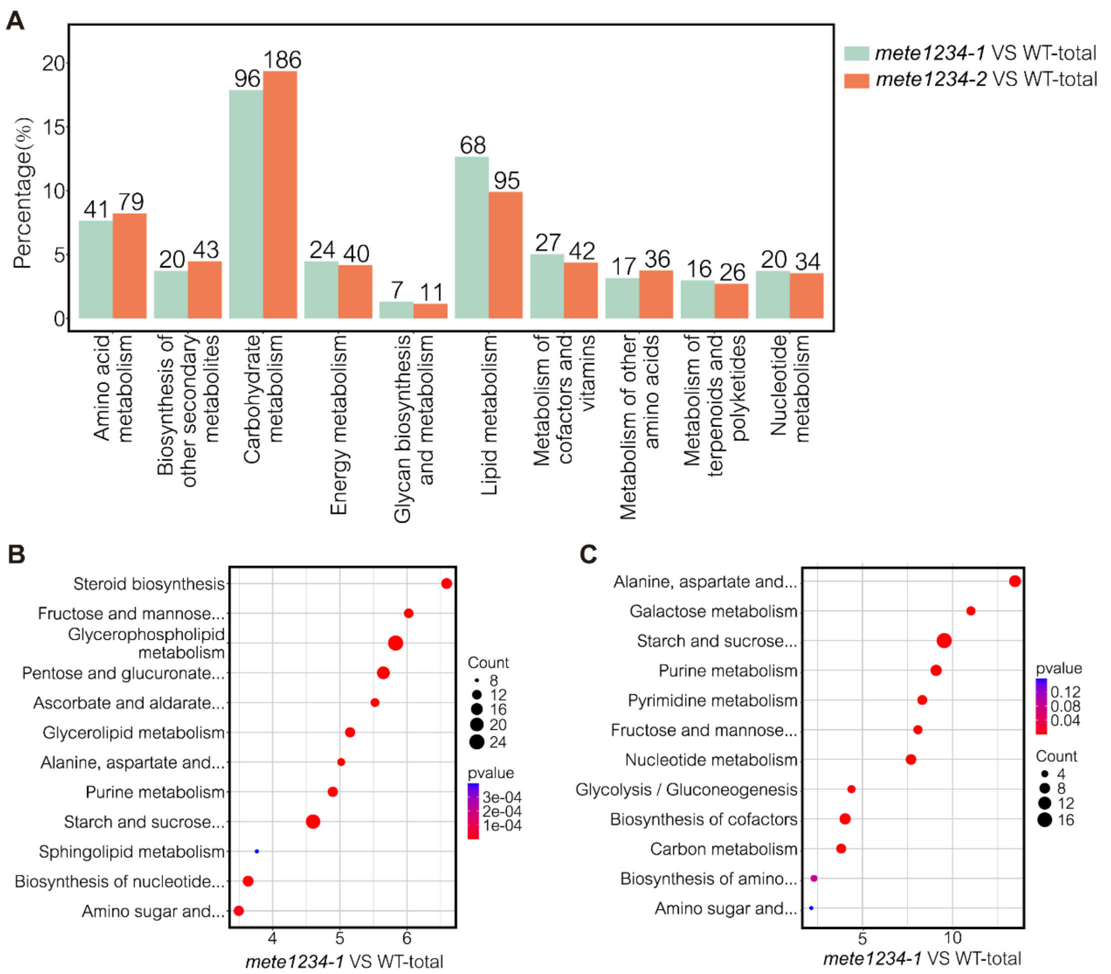

Supplementary Figure S7 GO and KEGG analysis of DEGs between the pollen of *mete1234* and WT

(A) Gene Ontology (GO) analysis of differentially expressed genes (DEGs) between pollen of *mete1234* and WT. **(B, C)** KEGG pathway enrichment analysis of upregulated DEGs in *mete1234* pollen compared to WT.

Supplemental Table S1. Anther or pistil abundantly expressed proteins identified by IP-LC-MS/MS

CL number	Uniprot accession	Mw (kD)	Protein name	Abbreviation
N000009	A0A1S4DH74	63	beta-fructofuranosidase, insoluble isoenzyme CWINV1-like	Beta-FFase
N000010	A0A1S3Z449	68	beta-glucosidase BoGH3B-like	BoGH3B
N000011	A0A1S4BXK3	53	Endoglucanase	EG
N000031	A0A1S3YQV4	23	HMG-Y-related protein B-like	HMG-Y
N000032	A0A1S3ZJV3	17	class I heat shock protein-like	sHSP
N000034	A0A1S3Z858	50	apyrase-like	APY
N000036	A0A1S4CZ78	40	polygalacturonase inhibitor-like	PGIP
N000038	A0A1S3YNC4	40	Glutamine synthetase	GS
N000040	A0A1S4B7N5	70	polyphenol oxidase E chloroplastic-like	PPO
N000043	A0A1S3YVF2	65	polyphenol oxidase E chloroplastic	PPO
N000044	A0A1S3Y633	23	vicilin-like seed storage protein	SSP
N000047	P68173	50	Adenosylhomocysteinase	SAHH
N000049	A0A1S3ZYM9	80	5-methyltetrahydropteroyltriglutamate-homocysteine methyltransferase-like	MetE
N000050	A0A1S3ZAU1	22	glycine-rich RNA-binding protein-like	GRP
N000051	A0A1S4BDR4	50	acetolactate synthase small subunit 1 chloroplastic like	ALS
T07	Q9M7K8	48	S-adenosylmethionine synthase 1	SAMS1

Supplemental Table S2. The basic information and physicochemical characteristics of NtMetEs

Gene ID	Protein ID	Length(aa)	Abbreviation
LOC107797732	XP_016476141.1	765	NtMetE1
LOC107811094	XP_016491448.1	765	NtMetE2
LOC107763602	XP_016437579.1	765	NtMetE3
LOC107791827	XP_016469454.1	765	NtMetE4
LOC107768359	XP_016442967.1	765	NtMetE5
LOC107827442	XP_016510071.1	765	NtMetE6
LOC104108327	XP_009615630.1	765	NtomMetE1
LOC104119942	XP_009629870.1	765	NtomMetE2
LOC104111436	XP_009619432.1	765	NtomMetE3
LOC104088570	XP_009591564.1	829	NtomMetE4
LOC104249971	XP_009804811.1	765	NsylMetE1
LOC104237549	XP_009790020.1	765	NsylMetE2
LOC104238725	XP_009791486.1	765	NsylMetE3
LOC104224931	XP_009774960.1	830	NsylMetE4

Supplemental Table S3. The sequence similarity of NtMetEs

Protein	NtMetE1	NtMetE2	NtMet3	NtMetE4	NtMetE5	NtMetE6
NtMetE1	100.00					
NtMetE2	98.82	100.00				
NtMetE3	95.82	95.16	100.00			
NtMetE4	96.21	95.56	98.43	100.00		
NtMetE5	95.95	95.56	96.34	95.95	100.00	
NtMetE6	95.82	95.56	96.21	95.82	99.35	100.00

Supplemental Table S4. The edited sequence of the mutants

Mutant	Sequence （5'→3'）	Type of mutation
mete23-1/2	*NtMetE2*：CCGGTG_TGAGATCGGATTCGAC	deletion of G base
	NtMetE3：CCAGTGAGTGAGATCGGGTTCG	insertion of A base
mete14-1/2	*NtMetE1*：CCGGTGAGTGAGATCGGATTCGAC	insertion of A base
	NtMetE4：CCGGTGAGTGAGATCGGGTTTGAC	insertion of A base
mete124-1/2	*NtMetE1*：CCGGTGAGTGAGATCGGATTCGAC	insertion of A base
	NtMetE2：CCGGTGAGTGAGATCGGATTCGAC	insertion of A base
	NtMetE4：CCGGTGAGTGAGATCGGGTTTGAC	insertion of A base
mete134-1/2	*NtMetE1*：CCGGTGAGTGAGATCGGATTCGAC	insertion of A base
	NtMetE3：CCAGTGAGTGAGATCGGGTTCG	insertion of A base
	NtMetE4：CCGGTGAGTGAGATCGGGTTTGAC	insertion of A base
mete12^{+-}*34-1/2*	*NtMetE1*：CCGGTGAGTGAGATCGGATTCGAC	insertion of A base
	NtMetE2-1：CCGGTGCGTGAGATCGGATTCGAC	insertion of C base
	NtMetE2-2：CCGGTG____AGATCGGATTCGAC	deletion of GTG base
	NtMetE3：CCAGTGAGTGAGATCGGGTTCG	insertion of A base
	NtMetE4：CCGGTGTGTGAGATCGGGTTTGAC	insertion of T base
mete1234-1/2	*NtMetE1:*CCGGTGAGTGAGATCGGATTCGAC	insertion of A base
	NtMetE2：CCGGTGCGTGAGATCGGATTCGAC	insertion of C base
	NtMetE3：CCAGTGAGTGAGATCGGGTTCG	insertion of A base
	NtMetE4-1：CCGGTGTGTGAGATCGGGTTTGAC	insertion of T base
	NtMetE4-2：CCGGTG_TGAGATCGGGTTTGAC	deletion of G base

target1:CCCATCTAGATACAACTGGACCG
target2:CCGGTGGTGAGATCGGATTCGAC

Supplemental Table S7. Primers used to explore the subcellular localization of *NtMetEs*

Usage	Primer name	Sequence（5'→3'）
Primers to clone *NtMetEs* into pBWA(V)HS-GLosgfp	*NtMetE1*-SF	CAGTCGTCTCA/CAACATGGCATCTCACATAGTTGG
	NtMetE1-SR	CAGTCGTCTCA/TACACTTGGCGCTGGCAAGCTGGG
	NtMetE2-SF	ATTTGGAGAGGACAGGGTACC/ATGGCATCTCACATAGTTGGAT
	NtMetE2-SR	GGCAGCAGCTCTAGAGGATCC/AACCATGGCTTGGAGTGC
	NtMetE3-SF	ATTTGGAGAGGACAGGGTACC/ATGGCATCTCACATTGTTGGAT
	NtMetE3-SR	GGCAGCAGCTCTAGAGGATCC/CTTGGCACTGGCAAGCTGA
	NtMetE4-SF	ATTTGGAGAGGACAGGGTACC/ATGGCATCTCACATTGTTGGATACC
	NtMetE4-SR	GGCAGCAGCTCTAGAGGATCC/AACTCTATCGGCTATCTCCTCTGTG
	NtMetE5-SF	CCTTCAGTTTCGCGAGCTCGGTACC/ATGGCATCTCACATTGTTGGATACC
	NtMetE5-SR	TTGCTCACCATGGTTCTAGAGGATCC/AACTCTATCGGCTATCTCCTCTGTG
	NsylMetE4-Ndel-SF	ATTTGGAGAGGACAGGGTACC/ATGGCATCTCACGTTGTTGGA
	NsylMetE4-Ndel-SR	GGCAGCAGCTCTAGAGGATCC/CTTGGTGCTGGCCAAGTGT
	NtCBL-SF	ATTTGGAGAGGACAGGGTACC/ATGTCATATTCTGTGTCTCTCAAGCAG
	NtCBL-SR	GGCAGCAGCCTAGAGGATCC/TGCTGGCCCAGTCCTGA
	NtSAHH-SF	ATTTGGAGAGGACAGGGTACC/ATGGCTCTATTAGTCGAGAAGACCA
	NtSAHH-SR	GGCAGCAGCTCTAGAGGATCC/GTACCTGTAGTGAGCAGGCTTG
	NtSAM-SF	ATTTGGAGAGGACAGGGTACC/ATGGAAACTTTCTTGTTCACCTCAGA
	NtSAM-SR	GGCAGCAGCTCTAGAGGATCC/AGCTTTTGGCTTGAGGACCT

Note: Underlined nucleotides indicate enzyme cleavage sites.

Supplemental Table S8. Primers used to detect the expression levels of *NtMetEs* by RT-qPCR

Primer name	Sequence（5'→3'）
NtMetE1-F	TTCTCTTTGCCCACGTTCATATAC
NtMetE1-R	ACTGCTCTTTCCATCCCAGAA
NtMetE2-F	GCCACGTTCAATATAGTTCTCTTT
NtMetE2-R	ATCCCAGAAAGATTCGAGAGCA
NtMetE3-F	TGTTGTTGTTTCTTCGCCAAT
NtMetE3-R	ACCTTCTTCAAGTCCTCAGCG
NtMetE4-F	TGGTCCTCAGCTGCTCAAAG
NtMetE4-R	AGGTCTCAAACCTTGGCTGG
NtMetE5-F	CTGAGGTAAAGCCAGCCCTC
NtMetE5-R	CCCACGCCTTCAATCTGACT
NtMetE6-F	GAAGGAGTCGCGACATATCA
NtMetE6-R	GGGCAGAGCTAACCTACACA
Ntubc2-F	CTGGACAGCAGACTGACATC
Ntubc2-R	CAGGATAATTTGCTGTAACAGATTA

Supplemental Table S9. Primers and targets for constructing the *NtMetEs* mutants by CRISPR-Cas9

	Primer name	Sequence（5'→3'）
Targets (red) and their cloning primers	sgRNA-1-F	CAGTGGTCTCA/TGCACGGTCCAGTTGTATCTAGAT
	sgRNA-1-R	CAGTGGTCTCA/AAAC**ATCTAGATACAACTGGACCG**
	sgRNA-2-F	CAGTGGTCTCATGCAGTCGAATCCGATCTCACCAC
	sgRNA-2-R	CAGTGGTCTCAAAAC**GTGGTGAGATCGGATTCGAC**
	M13-F	CCAGAAATTGAACGCCGAAG
	zmpl-R	GTAAAACGACGGCCAGT
Primers for mutants identification	*NtMetE1*-MF	AAGAATGGATCTTTACTAGTATG
	NtMetE1-MR	GGATCAAATAGTTATGATCAGTATG
	NtMetE2-MF	TCTGGAGTTATAGATTTGATCG
	NtMetE2-MR	ATCAAATAGTGATGATCAGTATG
	NtMetE3-MF	CAAACGCGAGCTAAGTATT
	NtMetE3-MR	GTATACATAAGAAAAGAATCACATA
	NtMetE4-MF	GTGTGAATAAAGAATGAGTTTAAG
	NtMetE4-MR	ATCAGTCTTTCTAGAGTCTAGA
